# Deciphering the microbial and molecular responses of geographically diverse *Setaria* accessions grown in a nutrient-poor soil

**DOI:** 10.1371/journal.pone.0259937

**Published:** 2021-12-08

**Authors:** Matthew J. Peterson, Pubudu P. Handakumbura, Allison M. Thompson, Zachary R. Russell, Young-Mo Kim, Sarah J. Fansler, Montana L. Smith, Jason G. Toyoda, Rosey K. Chu, Bryan A. Stanfill, Steven C. Fransen, Vanessa L. Bailey, Christer Jansson, Kim K. Hixson, Stephen J. Callister

**Affiliations:** 1 Environmental Molecular Sciences Laboratory, Pacific Northwest National Laboratory, Richland, Washington, United States of America; 2 Department of Soil and Crop Sciences, Texas A&M University, College Station, Texas, United States of America; 3 Biological Sciences Division, Pacific Northwest National Laboratory, Richland, Washington, United States of America; 4 Applied Statistics and Computational Modeling, Pacific Northwest National Laboratory, Richland, Washington, United States of America; 5 Irrigated Agriculture Research and Extension Center, Washington State University, Prosser, Washington, United States of America; University of Hyderabad, INDIA

## Abstract

The microbial and molecular characterization of the ectorhizosphere is an important step towards developing a more complete understanding of how the cultivation of biofuel crops can be undertaken in nutrient poor environments. The ectorhizosphere of *Setaria* is of particular interest because the plant component of this plant-microbe system is an important agricultural grain crop and a model for biofuel grasses. Importantly, *Setaria* lends itself to high throughput molecular studies. As such, we have identified important intra- and interspecific microbial and molecular differences in the ectorhizospheres of three geographically distant *Setaria italica* accessions and their wild ancestor *S*. *viridis*. All were grown in a nutrient-poor soil with and without nutrient addition. To assess the contrasting impact of nutrient deficiency observed for two *S*. *italica* accessions, we quantitatively evaluated differences in soil organic matter, microbial community, and metabolite profiles. Together, these measurements suggest that rhizosphere priming differs with *Setaria* accession, which comes from alterations in microbial community abundances, specifically Actinobacteria and Proteobacteria populations. When globally comparing the metabolomic response of *Setaria* to nutrient addition, plants produced distinctly different metabolic profiles in the leaves and roots. With nutrient addition, increases of nitrogen containing metabolites were significantly higher in plant leaves and roots along with significant increases in tyrosine derived alkaloids, serotonin, and synephrine. Glycerol was also found to be significantly increased in the leaves as well as the ectorhizosphere. These differences provide insight into how C_4_ grasses adapt to changing nutrient availability in soils or with contrasting fertilization schemas. Gained knowledge could then be utilized in plant enhancement and bioengineering efforts to produce plants with superior traits when grown in nutrient poor soils.

## Introduction

*Setaria* is a geographically widespread genus of C_4_ grass that includes more than 100 species [1]. It is part of the larger Panicoideae subfamily that also includes important biofuel and commercial grain crops including maize, *Miscanthus*, switchgrass, sorghum, and others. Two model species of *Setaria*, *S*. *viridis* (common name: foxtail millet) and *S*. *italica* (common name: green foxtail) have been researched extensively to examine and understand abiotic and biotic stress and tolerance within C_4_ grasses [2, 3]. *S*. *viridis* is the wild ancestor of *S*. *italica* [4], and both exhibit drought and salt tolerance and optimized water-use efficiency, making them suitable for research pertaining to the cultivation of bioenergy crops in nutrient and water limited environments [5–13]. Additionally, *Setaria* in general are well-suited for growth chamber and laboratory research because of their optimal size and short generation time [14]. They also have a small, fully sequenced genome (~490 Mb) that enables high-throughput molecular analyses [15].

The characteristics that make *S*. *viridis* and *S*. *italica* important model C_4_ grasses extend to their ectorhizospheres as well. Here, the ectorhizosphere is defined as the zone immediately surrounding, and influenced by, plant roots [16]. Ectorhizosphere studies associated with *Setaria* have thus far focused on understanding nitrogen fixation and general microbial taxonomic structure. For example, Okon et al. (1983) inoculated *S*. *italica* rhizospheres with *Azospirillum brasilense*, a known nitrogen fixer, to understand how it would impact the growth of *Setaria*. They found that the bacteria did not directly transport fixed nitrogen to plants but rather facilitated nutrient transport via nitrogen mineralization [17]. A study conducted by Jin et al. (2017) assessed microbial communities in the rhizosphere of *S*. *italica* and determined that plants selectively recruited microbes through exudation [7].

The studies above are important examples contributing to our understanding of the microbial composition and function of the plant-microbe relationship of C_4_ grasses within horticultural, or uncharacterized agricultural soils. Less is known specifically about the microbial composition and function of *S*. *italica* and *S*. *viridis* ectorhizospheres within a nutrient poor soil. Further, how the microbial and molecular composition adjust with nutrient addition that often must take place for successful agricultural cultivation in such a soil. To our knowledge there has only been one reported study to investigate *Setaria* responses to nutrient addition in a nutrient-poor soil environment. Nadeem et al. (2018) investigated *S*. *italica* (L.) Beauv, variety Yugu 1, in response to a low nitrogen environment and found that the growth of roots was inhibited by this environment [18], molecular analyses were largely made on roots, but investigation of the ectorhizosphere in combination with the plant was not undertaken. Thus, it is not known how nutrient availability affects the corresponding ectorhizosphere and soil microbiome. Due to this knowledge gap, we chose to investigate how the *Setaria* ectorhizosphere responds to a nutrient poor soil receiving a nutrient addition (fertilizer).

We measured leaf, root and soil metabolite and soil organic matter (SOM) profiles as well as the ectorhizosphere microbial responses of three *S*. *italica* accessions from three divergent geographical origins (Afghanistan, China, and India) as well as the *S*. *viridis* reference genotype (A10.1). The geographically diverse origins of the accessions led us to question how their ectorhizospheres would resemble each other before and after nutrient addition. To address this question, all accessions were grown within the same growth chamber and in the same nutrient-poor soil with (WNA; with nutrient addition) or without (NNA; no nutrient addition) periodic nutrient addition (i.e., fertilizer). Analyses of SOM, metabolites, and microbial taxa on soil from no plant controls and bulk soil were included to help differentiate the impact of *Setaria* on the native soil microbiome. Our results primarily focused on comparing the ectorhizospheres of those *S*. *italica* accessions exhibiting the largest or smallest response to nutrient addition in the context of SOM, metabolites and microbial taxa. However, we also measured and evaluated metabolites that are differentially produced by *Setaria* tissues (leaf and root) when comparing WNA to NNA and what, if any, associations could be made between these metabolic profiles, ectorhizosphere microbiome and plant phenotypic changes. We also hypothesized that based upon the methodology used here to separate the ectorhizosphere from bulk soil, SOM and microbial differences between ectorhizospheres of *Setaria* accessions WNA would be discernable.

## Materials and methods

### Soil description

The soil used in this experiment was part of the Warden Series (USDA; https://soilseries.sc.egov.usda.gov/OSD_Docs/W/WARDEN.html) found within Southeastern Washington State, and was collected at the Washington State University Irrigated Agriculture Research and Extension Center, Prosser, WA. The series is classified as a coarse-silty, mixed, superactive, mesic Xeric Haplocambid. The soil was air dried and then sieved to 4 mm to homogenize the sample and remove any large debris. Soil characterization was done by Soil Test Farm Consultants, Inc. (Moses Lake, WA). The texture is a sandy loam with a ratio of 50% sand: 5% clay: 45% silt, and is slightly basic (pH 8.2). The elemental composition of the soil contains P (7 mg/kg), K (203 mg/kg), Bo (mg/kg), Zn (mg/kg), Mn (mg/kg), Cu (0.3 mg/kg), Fe (3 mg/kg), Ca (15 meq/100g), Mg 3.8 meq/100g), and Na (19.5 meq/100g). The saturated hydraulic conductivity is 5.47 x 10^−4^ cm/s (4.73 x 10^−1^ m/day). The nutrient composition consists of 0.4% of organic matter, 1.0 mg/kg of Ammonium-N, 8.2 mg/kg of Nitrate-N, and 17 mg/kg of Sulfate-S. For successful cultivation, nutrients in the form of commercial fertilizers are commonly applied to a soil with this nutrient profile.

### Growth of *Setaria*

Two *Setaria spp*. consisting of three *Setaria italica* and one *Setaria viridis* accessions were selected for this study. Three biological replicates of *Setaria viridis* (A10.1, designated as A1 in this study), *Setaria italica* from India (Pl633416, designated as A2), China (Pl633417, designated as A3), and Afghanistan (Pl207502, designated as A4) were grown for a total of 12 plant-soil systems, along with three ‘no plant’ soil controls. *Setaria* seeds were sterilized in 5% bleach for 3 minutes and rinsed in sterilized water. Seeds were germinated on sterilized moist filter paper for two days at 30°C (during the light cycle) and 22°C (during the dark cycle). Sieved soil was potted (n = 3), and germinated seeds were planted in 300 g of soil. Plants were grown at 16:8 hr, light:dark cycles, 30°C: 22°C temperatures, 50–60% relative humidity (RH), ~400 ppm CO_2_, and at ~350 μmol m^-2^ s^-1^ light intensity. Additional pots (n = 3), containing only 300 g of soil were retained as no plant controls. Pots were watered every other day with 50 mL of water. There were two treatments: fertilized (with nutrient addition; WNA), or unfertilized (no nutrient addition; NNA). Every 4^th^ day the fertilized treatment received 50 mL of 1:1 water: 100 ppm fertilizer mix, while the unfertilized plants did not receive any nutrient additions. The fertilizer mix used was Jack’s Professional Fertilizer^©^ at a ratio of 20% nitrogen, 20% phosphorus, and 20% potassium. This nutrient addition regimen resulted in a total of 6.9 μg Urea-N, 4.1 μg of Nitrate-N 2.6 μg of Ammonium-N, 13.9 μg of K_2_O, and 13.9 μg of P_2_O_5_ added to each gram of soil every 4 days during the duration of the experiment.

### Harvesting of soil and *Setaria*

Plants were harvested at 4 weeks and were split into above- and below-ground portions by cutting the plant stem at the soil surface. Above-ground tissues were then measured, weighed, and placed in a 50 mL falcon tube and were flash frozen in liquid nitrogen. Above-ground tissues were then freeze-dried in a lyophilizer and re-weighed for dry biomass measurements. Bulk soil was separated from the ectorhizosphere soil. Briefly, after cutting away the pot, soil that fell immediately away from the roots and from the roots after slightly tapping against a hard surface was considered bulk soil (adapted from [19]). Upon separation, bulk soil was collected in a 50 mL falcon tube, weighed, and flash frozen in liquid nitrogen. The remaining soil that was attached to the root complex (ectorhizosphere soil) was separated from the root by placing the root with attached soil into a 50 mL falcon tube that contained 20 mL of a 0.2 mM Calcium chloride dihydrate (CaCl_2_×2H_2_O) buffer (S1 Fig) [19]. Each tube was pulsed (vortexed) for three consecutive 45 s intervals, which separated the ectorhizosphere soil from the root. Roots were then removed from the buffer solution, washed in nanopure water, placed in a 50 mL falcon tube, and flash frozen in liquid nitrogen. Root samples were also freeze-dried in a lyophilizer and re-weighed for dry biomass measurements. Each tube containing the ectorhizosphere soil fraction was subsequently centrifuged for 20 min at 4,000 x g to create a soil pellet. The supernatant was separated into 15 mL falcon tubes, and flash frozen in liquid nitrogen for later TOC/TNb and FTICR analyses. Ectorhizosphere soil pellets were flash frozen in liquid nitrogen. All above- and below-ground samples were stored in a -80°C freezer. To generate the liquid supernatant for the bulk soil fractions, 10 g of bulk soil was placed into a 50 mL falcon tubes, 20 mL of CaCl_2_ × 2H_2_O buffer (0.2 mM) was put into each tube and then pulsed as described above (S1 Fig). Tubes were centrifuged for 20 min at 4,000 x g to create a soil pellet, and the supernatant was split into two 15 mL fractions, one for TOC/TNb analysis and one for FTICR analysis. All tubes were flash frozen and stored at –80°C.

### 16S and ITS rRNA sequencing

DNA was extracted from 300–380 mg of bulk and ectorhizosphere soil using a MoBio PowerSoil kit (Qiagen Germantown, MD, USA) according to the manufacturer’s instructions, and further purified and concentrated (5 ng/μL) using a Zymo Genomic DNA & Clean Concentrator kit (Zymo Research, Irvine, CA). DNA was quantified using a Synergy 2 Multi-Detection Microplate Reader (BioTek Instruments, Winoosky, VT) and NanoDrop Spectrophotometer (Thermo Fisher Scientific, Waltham, MA). PCR amplification of the V4 region of the 16S rRNA gene and ITS (Internal Transcribed Spacer) was performed using the protocol developed by the Earth Microbiome Project (http://press.igsb.anl.gov/earthmicrobiome/emp-standard-protocols/16s/), and described in Caporaso et al. (2012), with the exception that the twelve base barcode sequence was included in the forward primer [20]. Amplicons were sequenced on an Illumina MiSeq using the 500 cycle MiSeq Reagent Kit v2 (http://www.illumina.com/) according to manufacturer’s instructions. An internal standard consisting of *Penicillium chrysogenum* DNA was added as a quality control. Reads were processed using PNNL’s in-house developed software tool, hundo, according to Brown et al. (2018) [21]. Briefly, 16S and ITS sequence data annotation was completed via hundo. Paired-end sequences were quality trimmed and filtered using BBDuk 2 and were then merged and dereplicated using VSEARCH. Merged reads were then clustered into operational taxonomic units (OTUs) with 97% similarity. SILVA [22, 23] was used as the annotation reference database for 16S data and UNITE [24, 25] was used for ITS data. Alignment of OTUs to taxonomic assignments via BLAST [26]. OTU counts were assigned using the global alignment method of VSEARCH to obtain an OTU table and biome table. Please see Brown et al. (2018) for detailed processing of read data [21]. Processed read data were analyzed in RStudio using package pmartR/pmartRseq [27] to remove sequence outliers, normalize counts, and calculate diversity metrics (Chao1, ACE, Jaccard index).

### Measurement of TOC and TNb

Total organic carbon and bound total nitrogen (TOC/TNb) within the collected ectorhizosphere and bulk soil supernatants was measured using a Vario TOC Combustion Analyzer (Elementar, Langenselbold, Hesse, de). Thawed samples (kept on ice) were diluted in acidified water (2:8 dilution by volume; 2 ml of sample and 8 ml of acidified water). The acidified water was prepared by adding 8.2 mL of concentrated HCl to 2 L of nanopure water. Seven variable volume standard samples (0.150 mL, 0.225 mL, 0.375 mL, 0.600 mL, 0.750 mL, and 1.5 mL) were generated by the instrument via dilution in acidified water of a 0.2 mM of CaCl_2_ ×2H_2_O buffer and 100 ppm carbon (C_8_H_5_KO_4_) + 50 ppm nitrogen (KNO_3_) working solution. Ectorhizosphere and bulk soil samples were analyzed in triplicate using the instrument TOC/TNb Precise method, that allows the instrument to calculate and then determine the volume of sample to inject to stay within the standard curve. Area measurements for each sample (along with blanks), provided by the instrument, were compared to standard curves (R^2^ = 0.99) to calculate ppm of C and N, that were then converted to mass (μg) and normalized by the soil dry weight (g) (S1 Table) which was obtained by weighing each soil, placed within an aluminum tin, before and after drying within an ~100°C oven.

### FTICR-MS soil organic analysis

Fourier transform ion cyclotron resonance mass spectrometry (FTICR-MS) was used to infer difference in dissolved soil organic matter (SOM) profiles among our samples [28]. We focused on polar solvent extraction of SOM based upon protocols that have been previously described [28], with the exception that prior to extraction, ectorhizosphere and bulk soil supernatants were adjusted to a common concentration of 20 ppm according to TOC concentrations (Table A in S1 Table) measured above. Solid-phase extraction clean-up of SOM consisted of using a PPL Bond Elut cartridge and following the procedure outlined in previous literature [29]. We used H_3_PO_4_ for acidification of the sample to a pH of 2 (checked with pH strips) and then rinsed the samples with 15 mL of 0.1 M HCl. We dried the cartridge using N_2_. Samples were eluted with 1.5 mL methanol (MeOH) with a total yield of about 1 mL. After clean-up, samples were directly infused at a flow rate of 3.0 μL/min into a 12 Tesla Bruker SolariX (Bruker SolariX, Billerica, MA) FTICR-MS outfitted with a standard electrospray ionization (ESI) interface and operated in negative mode. Instrument details have been previously described [28]. However, in brief, the MS instrument consisted of a home built automated Pal Autosampler (HTX technologies) coupled with Agilent 1200 series pumps (Agilent Technologies, Santa Clara, CA). Experimental conditions were as follows: needle voltage, +4.4 kV; Q1 set to 50 *m/z*; and the heated resistively coated glass capillary operated at 180°C. Data were collected by co-adding 144 scans from 100 *m/z* to 900 *m/z* at 4M and an ion accumulation time of 0.1 s.

One hundred forty-four individual scans were averaged for each sample and internally calibrated using an organic matter homologous series separated by 14 Da (–CH2 groups). The mass measurement accuracy was less than 1 ppm for singly charged ions across a broad *m/z* range (100–900 *m/z*). Data Analysis software (BrukerDaltonik version 4.2) was used to convert raw spectra to a list of *m/z* values applying FTMS peak picker module with a signal-to-noise ratio (S/N) threshold set to 7 and absolute intensity threshold to the default value of 100. Chemical formulae were then assigned using in-house software (Formularity, [30]) following the Compound Identification Algorithm (CIA), proposed by Kujawinski and Behn (2006), modified by Minor et al. (2012), and described in Tolic et al. (2017) [30–32]. Chemical formulae were assigned based on the following criteria: S/N >7, and mass measurement error < 0.5 ppm, taking into consideration the presence of C, H, O, N, S and P and excluding other elements. Assigned chemical formula observed within only one biological replicate per treatment (WNA or NNA) where discarded. Chemical formula approximated to chemical classes were based upon calculated oxygen to carbon and hydrogen to carbon ratios [33, 34].

### Root and leaf metabolomics analysis

Below- and above-ground portions of plant tissue were lyophilized and pulverized in a Geno/Grinder (SPEX, Metuchen, NJ) at 1,700 rpm for 15 minutes, then ~1.5 mL volume-sized replicates of plant powder were placed into 15 mL falcon tubes and weighed. Lyophilized rhizosphere and bulk soil samples were separated into 200 mg replicate soil pellets and placed into 2 mL microfuge tubes which were then weighed. All plant tissue and soil samples were stored at -80°C until ready for metabolite extractions.

Metabolites were extracted using a modified version of previously reported analyses [35, 36]. Modified here, a ratio of 2 mL of MeOH, 1.8 mL of chloroform, and 2 mL of nanopure water (2:1.8:2) was used in a volume which was 5x the volume of soil or plant material used in the extraction, for the separation of metabolites in the plant and soil samples. Samples were vortexed to create an emulsion followed by centrifugation at 5,700 x g for 5 minutes at 4°C to create two immiscible solvent layers and an insoluble protein pellet layer, which separated metabolites (water/methanol layer) from lipids (chloroform layer) and proteins (interphase). Extracted metabolites were pipetted out into 2 mL automatic liquid sample (ALS) vials which were placed under a nitrogen stream until dry. Dried metabolites were weighed and then re-suspended in a 50:50 mix of methanol and nanopure water, all at a concentration of 20 mg/mL as to normalize the total sample amount derivatized and loaded for each gas chromatography mass spectrometry (GC-MS) instrument run. One hundred μLs of each sample were transferred to new ALS vials and were again dried under a nitrogen stream prior to derivatization for GC-MS analysis. Individual biological root replicates of each accession did not provide enough metabolite mass individually as to be analyzed in a comparative fashion with the other samples so were combined into a single run for comparison to the triplicate analysis of the WNA root samples. Therefore, only a combined accession volcano plot comparison of the root samples could be performed.

An Agilent 7890A gas chromatograph coupled with a single quadrupole 5975C mass spectrometer (Agilent Technologies, Inc.) was used for all analyses. Metabolite extracts were dried *in vacuo* again to remove any residual moisture and were reconstituted in 5 μL pyridine containing methoxyamine, for methoxyamination of reactive carbonyl groups. To derivatize hydroxyl and amine groups to trimethylsilyated (TMS) forms, *N*-methyl-*N*-(trimethylsilyl)trifluoroacetamide (MSTFA) with 1% trimethylchlorosilane (TMCS) (80 μL) was added to each vial, followed by incubation at 37°C with shaking for 30 min. The samples were allowed to cool to room temperature and were analyzed on the same day. A HP-5MS column (30 m × 0.25 mm × 0.25 μm; Agilent Technologies) was used for untargeted analyses. Samples (1 μL) were injected in splitless mode, and the helium gas flow rate was determined by the Agilent Retention Time Locking function based on analysis of deuterated myristic acid (Agilent Technologies, Santa Clara, CA). The injection port temperature was held at 250°C throughout the analysis. The GC oven was held at 60°C for 1 min after injection, and the temperature was then increased to 325°C by 10°C/min, followed by a 5 min hold at 325°C. Data were collected over the mass range 50–550 *m/z*. A mixture of fatty acid methyl esters (C8–C28) was analyzed together with the samples for retention index alignment purposes during subsequent data analysis [37]. Samples were randomized and GC-MS measurements were deconvoluted, chromatographically aligned, and matched to an in-house developed metabolite spectral/retention index reference library using MetaboliteDetector [38]. Metabolite abundances obtained from each sample were transformed to a log_2_ value and these values were then normalized using a mean centering algorithm available in InfernoRDN [39]. Analysis of Variance (ANOVA) using a Welch approximation variance assumption was used to compare treatment means and biological replicates (n = 3).

## Results

### Response to nutrient addition varied with accession

**Fig 1:**
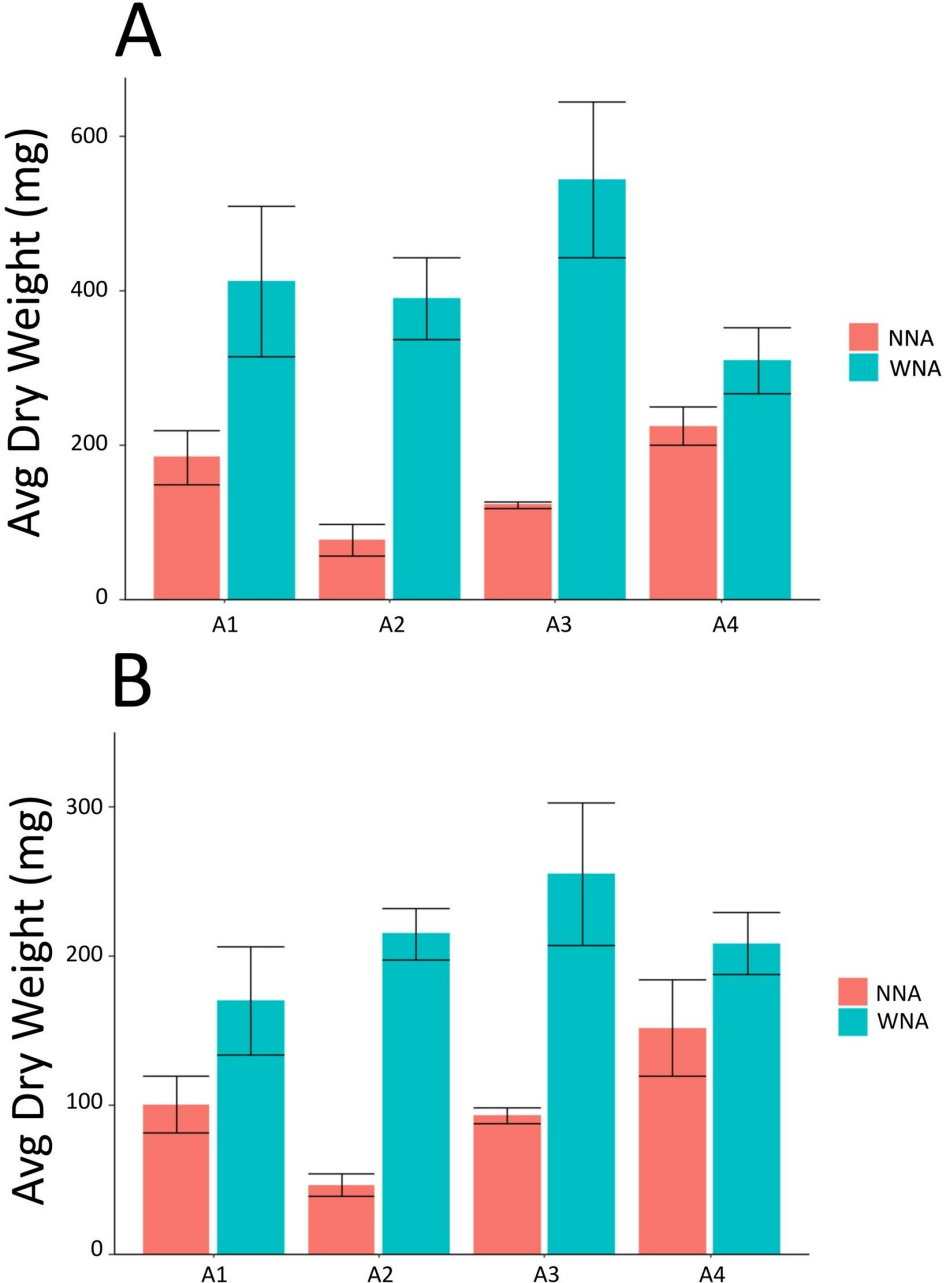
Average dry weight of *Setaria* **(A)** aboveground biomass and **(B)** belowground biomass with nutrient addition (WNA) and no nutrient addition (NNA).

The growth response of *Setaria* to nutrient addition (WNA) varied with accession as measured by above ground biomass, below ground biomass (Fig 1) and total biomass (S2 Fig). Significantly greater above-ground biomass was observed relative to the no nutrient addition control (no nutrient addition; NNA) for A2 (p < 0.01, WNA Mean = 389.90 mg ± 91.64, NNA Mean = 76.26 mg ± 34.33, expressed as dry weight) and A3 (p < 0.05, WNA Mean = 543.13 mg ± 175.14, NNA Mean = 121.73 mg ± 7.37) (Fig 1A). We also observed significantly greater below-ground biomass for A2 (p < 0.01, WNA Mean = 214.80 mg ± 29.55, NNA Mean = 46.13 mg ± 13.32) and A3 (p < 0.05, WNA Mean = 255.16 mg ± 83.00, NNA Mean = 92.53 mg ± 9.06) (Fig 1B). Compared to the other accessions, A4 exhibited the smallest growth response WNA, where the difference in biomass was not statistically significant in either the above or below ground biomass measurements. In contrast, A3 exhibited the largest difference in above ground growth response WNA.

**Fig 2:**
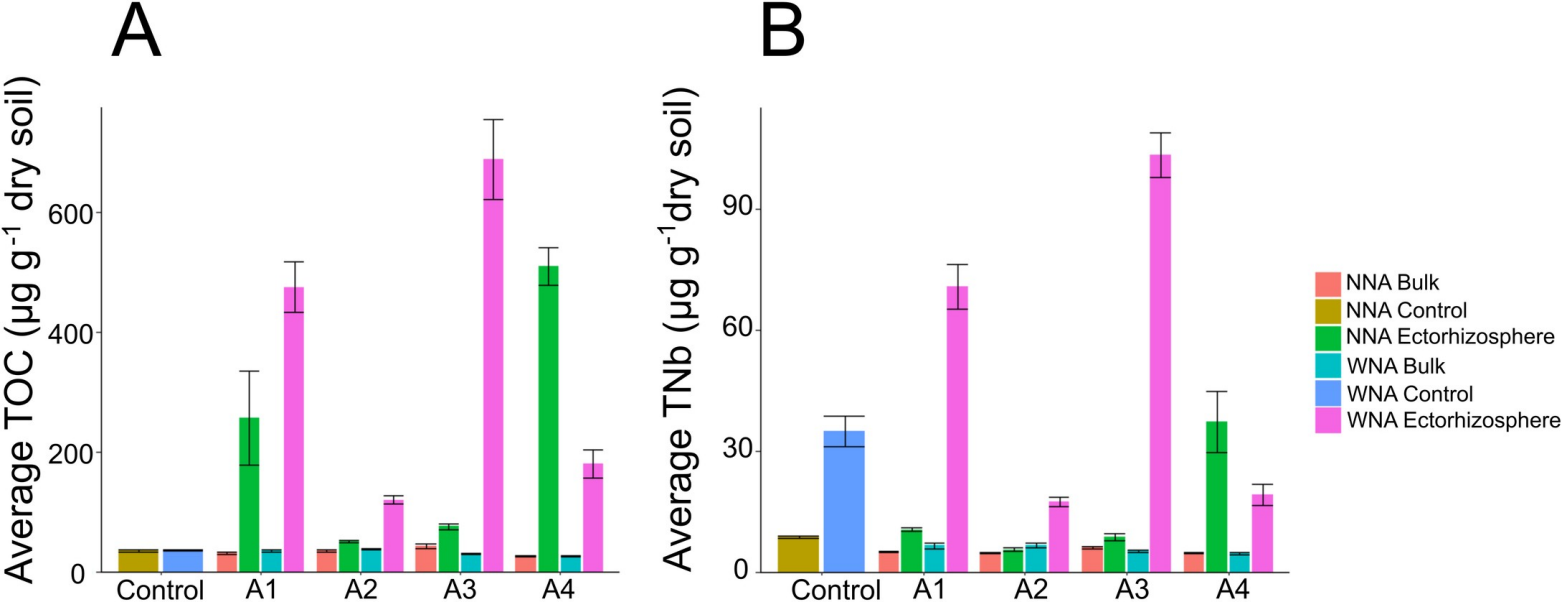
Measurement of **(A)** total organic carbon (TOC) and **(B)** total bound nitrogen (TNb) within soil water extracts amongst all *Setaria spp*. and accessions with nutrient addition (WNA) or no nutrient addition (NNA) in their respective soil. Data were normalized by dry weight of each harvested soil.

Total organic carbon (TOC) found within the soil water extractable fractions (see Methods-Harvesting), and expressed as mass per dry weight of soil, was significantly different (Welch’s ANOVA as part of InfernoRDN [3]) for the ectorhizosphere of all *Setaria* when compared to the NNA controls (p < 0.05), with the exception of A2 (Fig 2A). In contrast to the ectorhizospheres of A1 and A3, which showed significantly greater mass of TOC WNA compared to NNA, A4 exhibited the opposite; greater amounts of TOC in the NNA treatment compared to the WNA treatment (p < 0.001, NNA Mean = 509.04 μg/g^-1^ ± 94.81, WNA Mean = 179.92 μg/g^-1^ ± 71.03) (Fig 2A). Nutrient addition had no measured impact on TOC for soil contained within the no plant controls. And, given that the bulk soil fractions harvested from the same pots used for *Setaria* also showed no significant difference in measured TOC suggested that we successfully isolated the ectorhizosphere soil fraction.

Likewise, in the ectorhizospheres of all accessions, the average mass of TNb, expressed as mass per dry weight of soil, was significantly different WNA compared to the NNA controls (p <0.05) (Fig 2B). As found in the TOC measurements, bulk soil fractions showed no significant difference in measured TNb across all samples (Fig 2B), which further confirmed that our measurements successfully represented isolated ectorhizosphere. As expected, an increase in nitrogen WNA was observed for the soil within the no plant control. Additionally, the mean nitrogen abundance in our no plant control was greater than that measured within the ectorhizospheres for A2 and A4 (Fig 2B), suggesting a net increased uptake by the plant. As with TOC, we observed a greater abundance of TNb in the NNA replicates of A4 (p <0.001, WNA 19.04 μg/g^-1^ ± 7.93, NNA 37.27 μg/g^-1^ ± 22.73) demonstrating a contrasting response of its ectorhizosphere compared to those of the other accessions.

Of the *Setaria* accessions selected, we observed the largest difference in response between NNA and WNA for A3 in measured above and below ground biomass, and with its ectorhizosphere, regarding bulk TOC and TNb measurements. A4 exhibited the smallest difference in biomass and the opposite response in its ectorhizosphere for TOC and TNb. Because of this contrast, we chose to focus our molecular and microbial analyses on A3 and A4, as described below.

### SOM composition differed between *Setaria* ectorhizospheres

FTICR-MS measured a combined total of 31,275 *m/z* features from all accessions representing extracted water-soluble soil organic matter (SOM). Molecular formulae were assigned to 36% of these *m/z* features (11,381 out of 31,275; Table B in S1 Table). When accounting for biological reproducibility, 8,488 of these 11,381 molecular formulae were observed across all biological replicates and accessions. From the total number of observed molecular formulae, differences between bulk soil and ectorhizosphere SOM were observed (S3 Fig), which suggests distinct SOM profiles between these two soil fractions.

**Fig 3:**
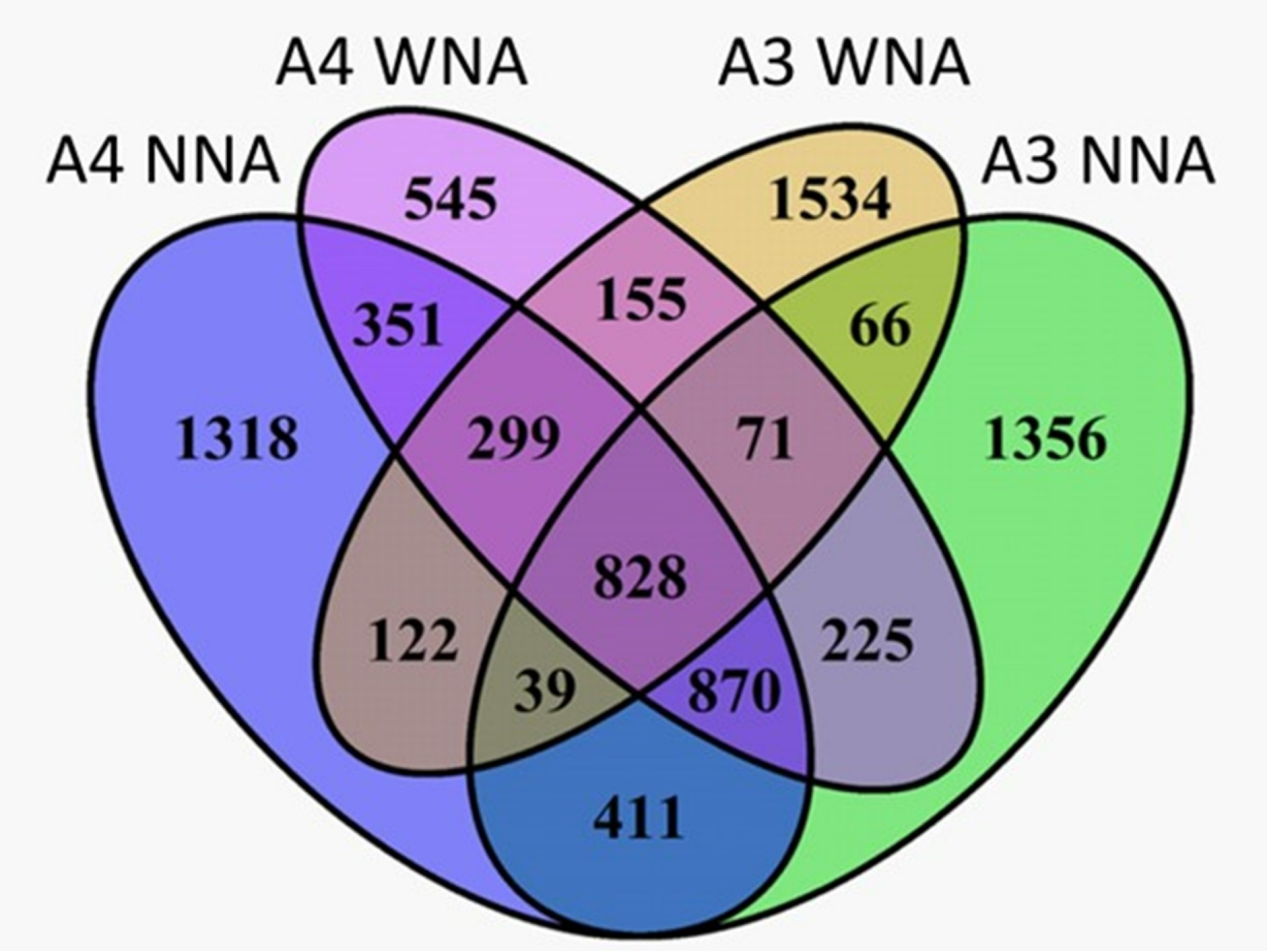
Four-way Venn diagram showing the number of uniquely identified chemical compounds between *Setaria italica* accessions A3 and A4 with nutrient addition (WNA) or no nutrient addition (NNA).

Reproducible SOM features measured by FTICR-MS for A1, A2, and A3 showed a high degree of overlap ranging from 90–98%, and A3 that exhibited the largest number of unique features of the three, was selected for comparison to A4. However, all SOM data for all *Setaria* is publicly available. When focusing on A3 and A4, we observed differences in the SOM chemical constituents, as expected due to the observed contrast in their ectorhizosphere responses measured for water extractable TOC and TNb (Fig 3). Here, 1,863 *m/z* features having molecular formulae were observed unique to A4, meaning these molecular formulae were measured within all biological replicates of A4, but were not measured within any of the biological replicates of A3. This value represents 22% of the total *m/z* features measured for A3 and A4 that were assigned molecular formulae. Further, 2,890 *m/z* features (35.25% of features between A3 and A4) having molecular formulae were observed unique to A3 when compared to A4 (Fig 3).

**Fig 4:**
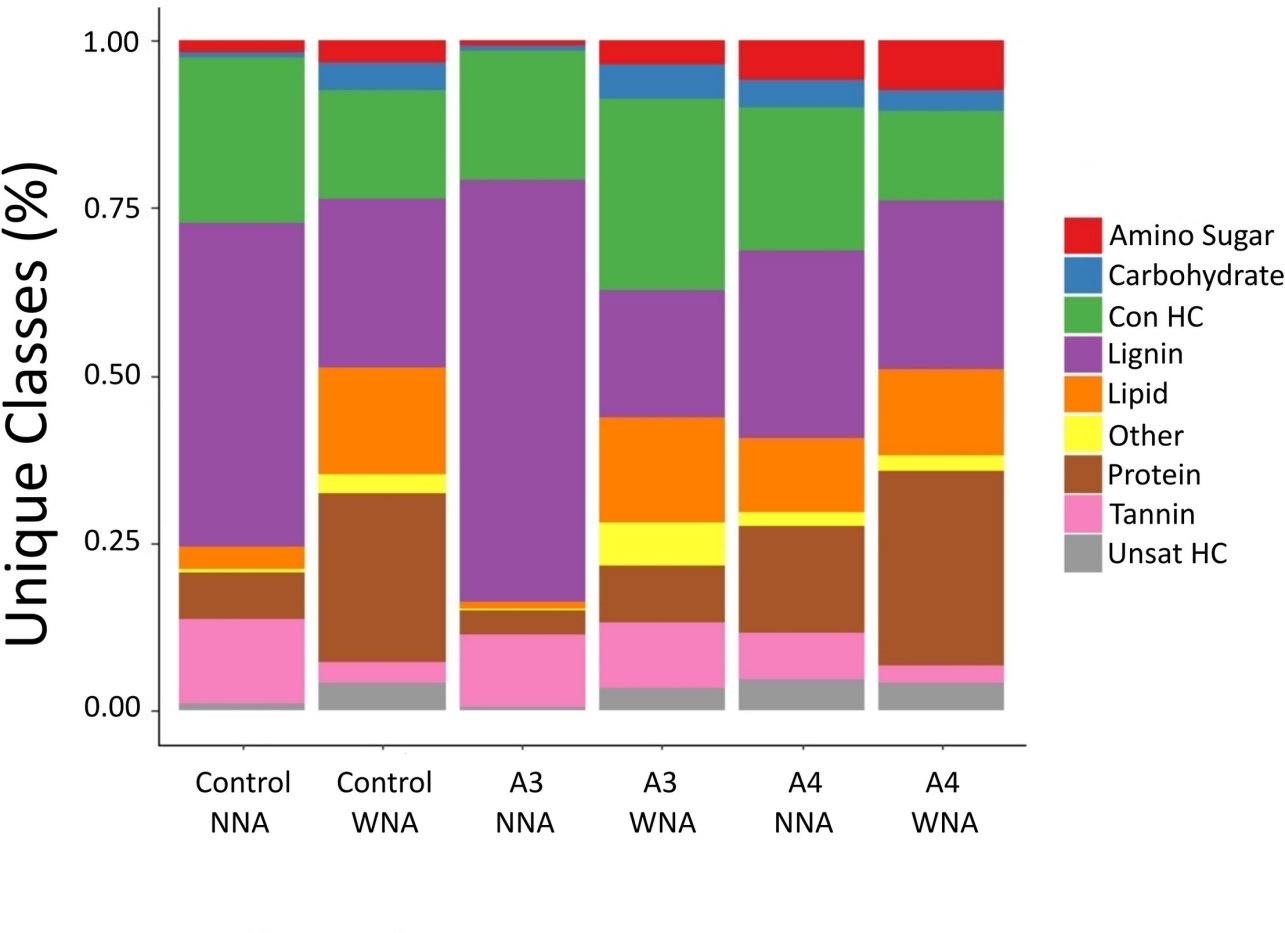
Stacked bar chart showing the percentage of FTICR-MS derived molecular formula assigned to unique chemical classes identified in the no plant control and ectorhizosphere soil samples. Ectorhizosphere data shown is for A3 and A4 *Setaria italica* accessions in the nutrient addition (WNA) and no nutrient addition (NNA) soil. In the legend, “Con HC” refers to condensed hydrocarbons, “Unsat HC” refers to unsaturated hydrocarbons.

The above SOM measurements provide evidence that the ectorhizospheres of A3 and A4 responded differently within the Warden soil to nutrient addition (Fig 3). To better characterize these molecular compositional differences, we assigned chemical classes to those uniquely measured FTICR-MS molecular formulae (Fig 4). Fig 4 summarizes this comparison by combining measurements from biological replicates and representing the number of chemical formulae within a class as a percentage of the total assigned chemical formulae for a treatment (WNA or NNA). On the other hand, Table 1 provides the mean percentage (by not combining biological replicates) of molecular formula making up a chemical class, standard deviation, and p-value results.

**Table 1:** Classification of soil organic matter (SOM) for *S*. *italica* accessions A3 and A4. Values represent mean percentage of FTICR-MS empirical features assigned to a given SOM class.

	Control		A3		A4	
	WNA	NNA	WNA	NNA	WNA	NNA
Amino Sugar	3.81 ± 0.43^ᵼ^ ^**ƍ**^	2.45 ± 0.46^ᵼ^	**8.17 ± 0.46** ^**ƍ**^	**4.21 ± 0.40**	10.25 ± 0.42^ᵼ^	8.40 ± 0.48^ᵼ^
Carbohydrate	0.72 ± 0.51^ᵼ^	0.47 ± 0.51^ᵼ^	**4.1 ± 0.37**	**1.62 ± 0.39**	4.03 ± 0.44^ᵼ^	4.51 ± 0.47^ᵼ^
Condensed Hydrocarbon	13.11 ± 0.47	16.87 ± 0.46	**15.14 ± 0.50**	**13.98 ± 0.45**	**9.26 ± 0.46**	**10.40 ± 0.47**
Lignin	50.45 ± 0.412^ᵼ^ ^**ƍ**^	52.11 ± 0.43^ᵼ^	**31.22 ± 0.49** ^**ƍ**^	**50.60 ± 0.45**	**39.27 ± 0.42** ^ᵼ^	**39.23 ± 0.49** ^**ᵼ**^
Lipid	5.70 ± 0.45	4.04 ± 0.47	**12.36 ± 0.44**	**4.51 ± 0.42**	7.55 ± 0.45	7.31 ± 0.44
Other	0.71 ± 0.48	0.37 ± 0.50^**ƍ**^	**2.77 ± 0.50**	**0.60 ± 0.42**	1.00 ± 0.48	1.10 ± 0.49^ᵼ^
Protein	15.09 ± 0.43	11.59 ± 0.43	**16.59 ± 0.48**	**12.54 ± 0.43**	18.97 ± 0.40	17.35 ± 0.44
Tannin	9.31 ± 0.41	11.54 ± 0.41	**7.90 ± 0.38**	**10.87 ± 0.44**	**7.65 ± 0.41**	**9.97 ± 0.44**
Unsaturated Hydrocarbon	0.97 ± 0.49^ᵼ^	0.56 ± 0.46	1.72 ± 0.50	1.06 ± 0.36	2.02 ± 0.48^ᵼ^	1.73 ± 0.43

Boldtext indicates significant differences between treatments, within accession (paired t-test, p < 0.01).

ᵼ indicates significant differences between A4 and Control (paired t-test, p < 0.05).

ƍ indicates significant differences between A3 and Control (paired t-test, p < 0.05).

The SOM WNA and NNA profiles for A4 are qualitatively alike regarding the fraction of molecular formulae assigned to a chemical class (Fig 4) and their means (Table 1). For example, lignin is the largest class for both WNA and NNA, comprising 39.3 ± 0.40 percent of total unique features and 39.2 ± 0.44 percent, respectively (Table 1), indicating no impact of treatment on this class. Yet, differences in some chemical classes were observed, namely the amino sugar class, and protein class, which increased in the above measures WNA. The percentages of measured molecular formula assigned to condensed hydrocarbons and tannins significantly decreased in A4 WNA (p < 0.001, respectively).

The SOM WNA and NNA profiles for A3 were observed as substantially different. The lignin class decreased WNA (Fig 4), and the mean percentage of the number of molecular formulae assigned to this class decreased (p <0.001) by 19 percent (from 50.6 ± 0.48 percent to 31.2 ± 0.39 percent; Table 1). As with A4, a decrease in the fractional response and mean percentage of tannin was observed with nutrient addition. The decrease in molecular formulae assigned to lignin and tannin and a systematic increase in formulae assigned to all the other SOM classes does not represent a systematic bias in the mass of carbon injected to the FTICR-MS, as all samples for analysis were adjusted to the same carbon concentration based upon our TOC measurements (Table A in S1 Table). Thus, for A3, the observed increase in TOC measured with nutrient addition is likely a result of an increase in several classes of organic matter offsetting lignin and tannin declines. The contrasting differences in SOM profiles for A3, A4, and the no plant control, especially with and without nutrient addition led us to hypothesize that a significant shift in microbiome composition would be observed.

### Ectorhizosphere microbial composition differed by population abundance and structure

**Fig 5:**
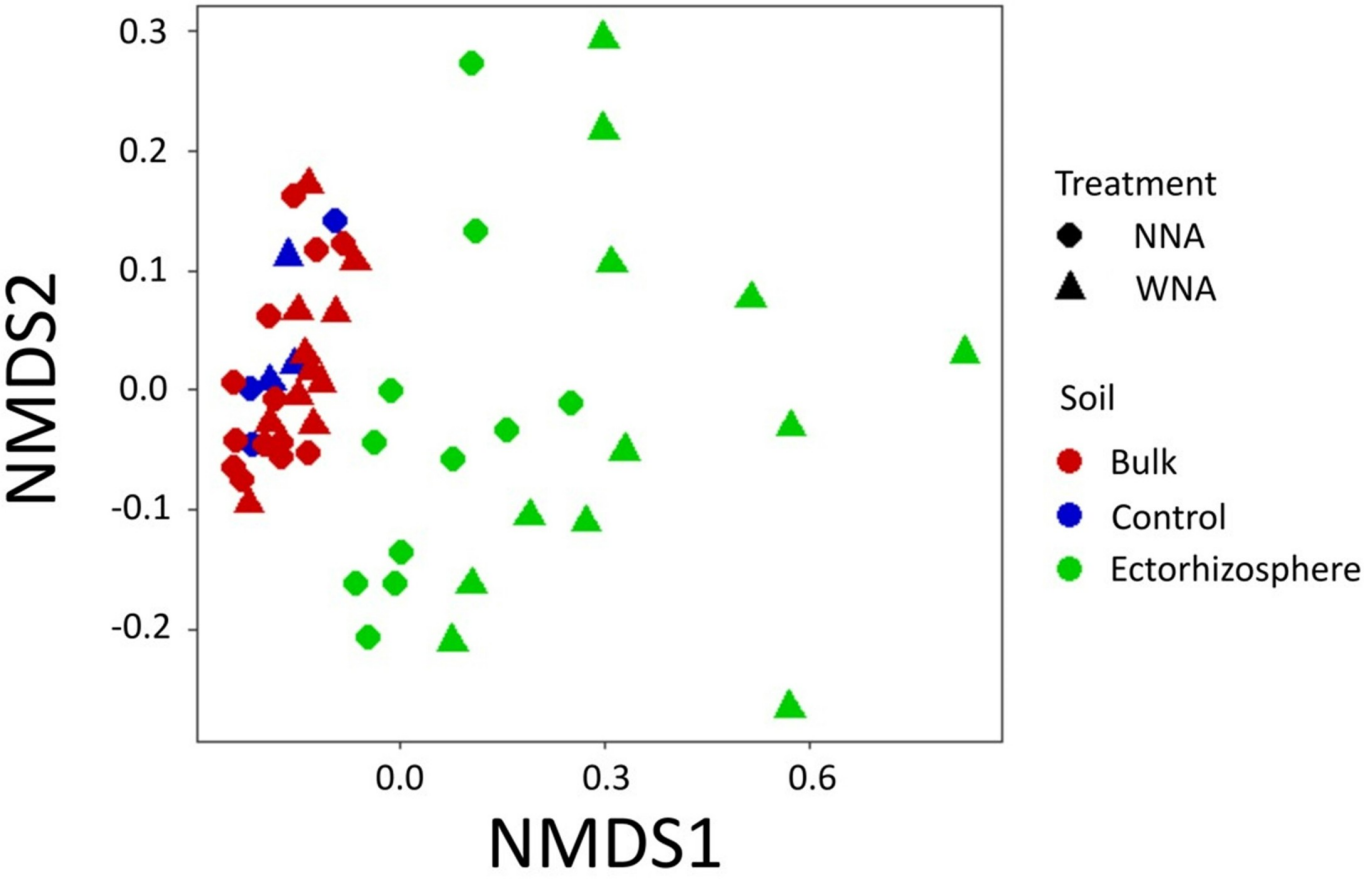
Non-metric multi-dimensional scaling (NMDS) plot of 16S sequence data acquired from with nutrient addition (WNA) or no nutrient addition (NNA) soils.

Nonmetric multi-dimensional scaling (NMDS) of bacterial OTUs measured by 16S rRNA amplicon sequencing separated *Setaria* ectorhizospheres generally from bulk soil samples (Fig 5). OTUs from no plant controls grouped with bulk soil OTUs demonstrating the influence of *Setaria viridis and Setaria italic* accessions (Fig 5) on surrounding ectorhizosphere soil, which has been previously demonstrated for different *Setaria spp*. [40]. Additionally, within the ectorhizosphere a division between nutrient addition and no nutrient addition was observed, providing evidence in favor of our hypothesis. Alpha-diversity (Shannon index) decreased WNA compared to NNA for A1, A2, and A3 but not for A4, where alpha-diversity remained observationally unchanged (S4 Fig). The decrease in diversity for *S*. *viridis* and these *S*. *italica* accessions was not statistically significant, even though abundance differences in major phyla were observed (S5 Fig). Therefore, to better delineate compositional differences, we constructed a 4-way Venn diagram to evaluate the contribution of uniquely observed taxa to nutrient addition focusing on A3 and A4. Of the 567 identified bacterial OTUs, 21 were observed unique to A3 (observed across all replicates in A3, but not in A4), and 59 were observed unique to A4 (S6 Fig). However, less delineation in OTU composition between A3 and A4 was observed with nutrient addition, where only 9 OTUs were observed as unique to A3, while 11 were observed as unique to A4 (S6 Fig). The classification of all OTUs can be found in Table C in S1 Table. Since the composition in the microbiome between A3 and A4 were observed to be largely conserved, we focused our analysis on differences in abundances between shared OTUs.

**Fig 6:**
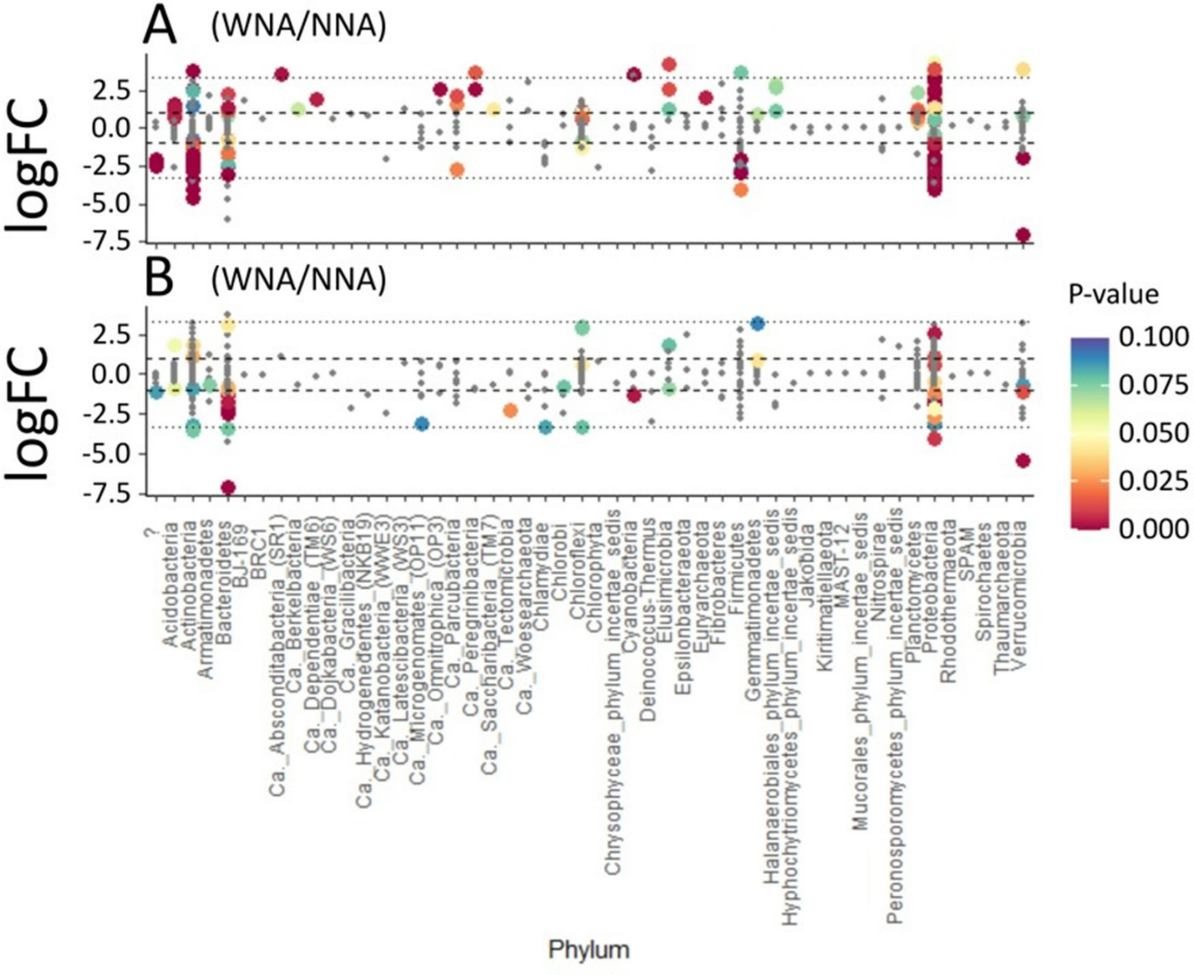
Fold-change of Log_2_ abundances, measured for bacterial OTUs assigned to phyla, comparing no nutrient addition (NNA) to with nutrient addition (WNA) for the ectorhizosphere harvested from *Setaria italica* accession A3 **(A)** and accession A4 **(B)**.

Fig 6 compares the abundances (using Log_2_ fold changes) of OTUs within each treatment that were found in both A3 and A4 at the phylum level. When comparing the effect of treatment within each accession on the abundance of OTUs we observed large abundance shifts in OTUs within Actinobacteria, Bacteroidetes, and Proteobacteria in both A3 and A4. Specifically, we observed more pronounced shifts in Actinobacteria and Proteobacteria abundances in accession A3 between the NNA and WNA treatment (Fig 6A). When comparing accessions, we observed that Actinobacteria and Proteobacteria had more significant (p <0.05) member shifts within A3. This suggests that A3 may have a stronger influence on the relative abundance of Actinobacteria and Proteobacteria (Fig 6A) than A4. However, A4 appears to influence the abundances of different members of mainly Bacteroidetes and Proteobacteria bacteria (Fig 6B). In general, we see larger differences in the relative abundances of OTUs associated with the ectorhizospheres of A3 than in A4.

**Fig 7:**
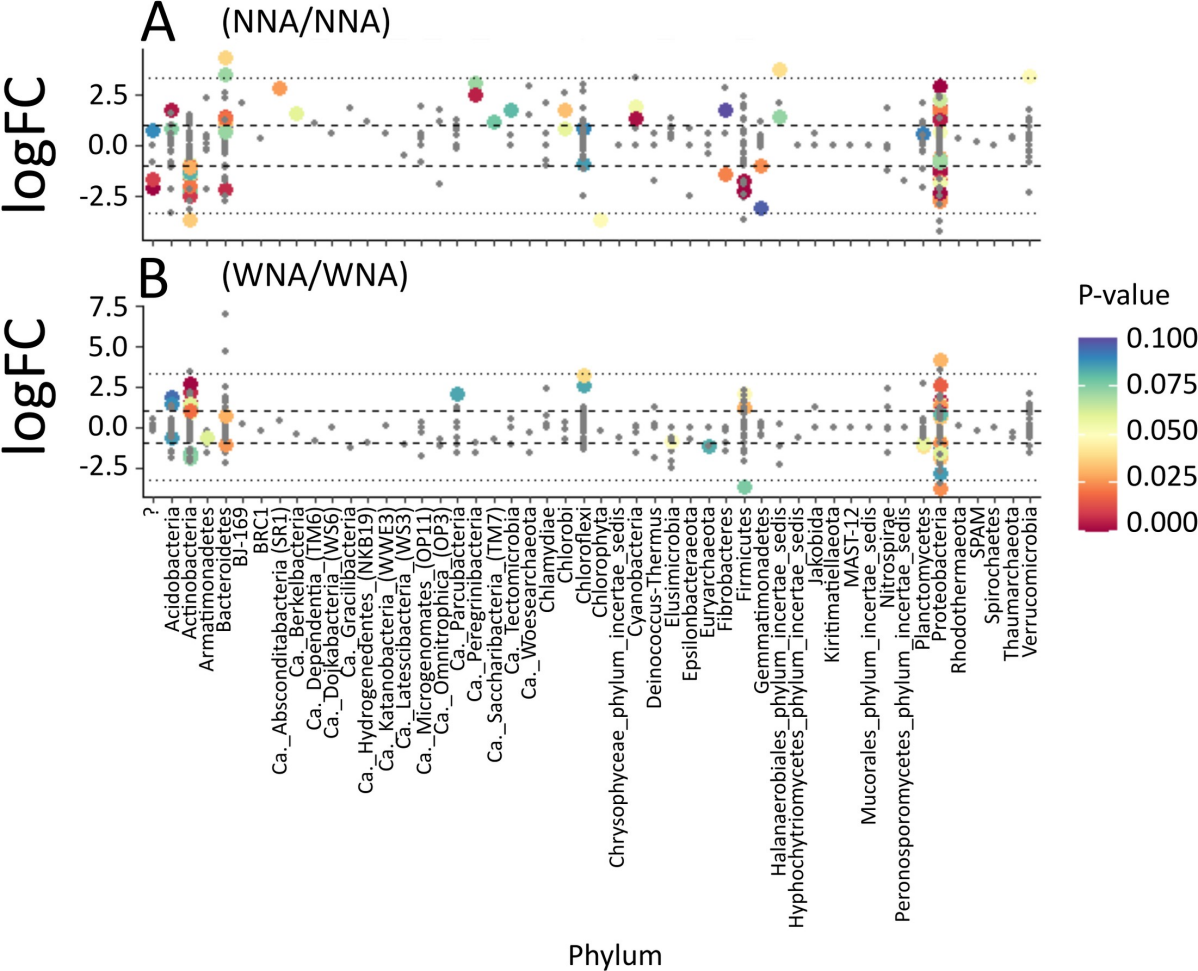
Fold-change of Log_2_ abundances, measured for bacterial OTUs assigned to phyla, comparing *Setaria italica* accession A3 to accession A4 for **(A)** no nutrient addition (NNA) and for **(B)** with nutrient addition (WNA) soils harvested from the ectorhizosphere.

While Fig 6 highlighted differences in bacterial OTUs between treatments, Fig 7 compares A3 and A4 within the same treatment to highlight bacterial abundance changes based on accession alone (Fig 7). The profiles of microbial phyla for A3 and A4 WNA are similar (Fig 7B), which was expected because of the similarity observed between A3 and A4 in both our TOC/TNb and FTICR-MS measurements. We observed changes in Actinobacteria abundance between accessions based on treatment. A3 shows a higher abundance of Actinobacteria in the WNA treatment (Fig 7B), while Actinobacteria was higher in abundance in the NNA treatment for A4 (Fig 7A). This qualitatively agrees with our observations in Fig 4, where lignin accumulates under NNA conditions but is significantly consumed under WNA conditions as Actinobacteria are well known for consuming lignocellulosic material as a substrate [41, 42]. When comparing A3 and A4 in the NNA treatment, A4 appears to impact the abundances of Actinobacteria, Bacteroidetes, and Firmicutes more than A3, while A3 appears to have greater impact on the abundances of Acidobacteria, Ca. Peregrinibacteria, and Cyanobacteria (Fig 7A). Proteobacteria was observed to be in high abundance in both the WNA and NNA treatments (Fig 7).

ITS amplicon sequencing measured fewer fungal OTUs in comparison to bacterial OTUs (S7 Fig). We observed the abundances of Ascomycota and Olpidiomycota OTUs to be significantly greater in A3 compared to A4 in the NNA treatment (S8 Fig). Also, in the NNA treatment, OTUs associated with Basidiomycota were measured as significantly greater in abundance within A4 compared to A3 (S8 Fig). When comparing A3 to A4 in the WNA treatment, A3 showed almost no effective influence on ectorhizosphere fungal OTUs. Few, if any, fungal OTUs were observed in any abundance in A3 WNA (S9 Fig). Conversely, A4 shows a significant increase in Ascomycota and Olpidiomycota OTU abundances (S9 Fig).

### Metabolite enrichment in leaf, root, and ectorhizosphere metabolites differed with nutrient addition

**Fig 8:**
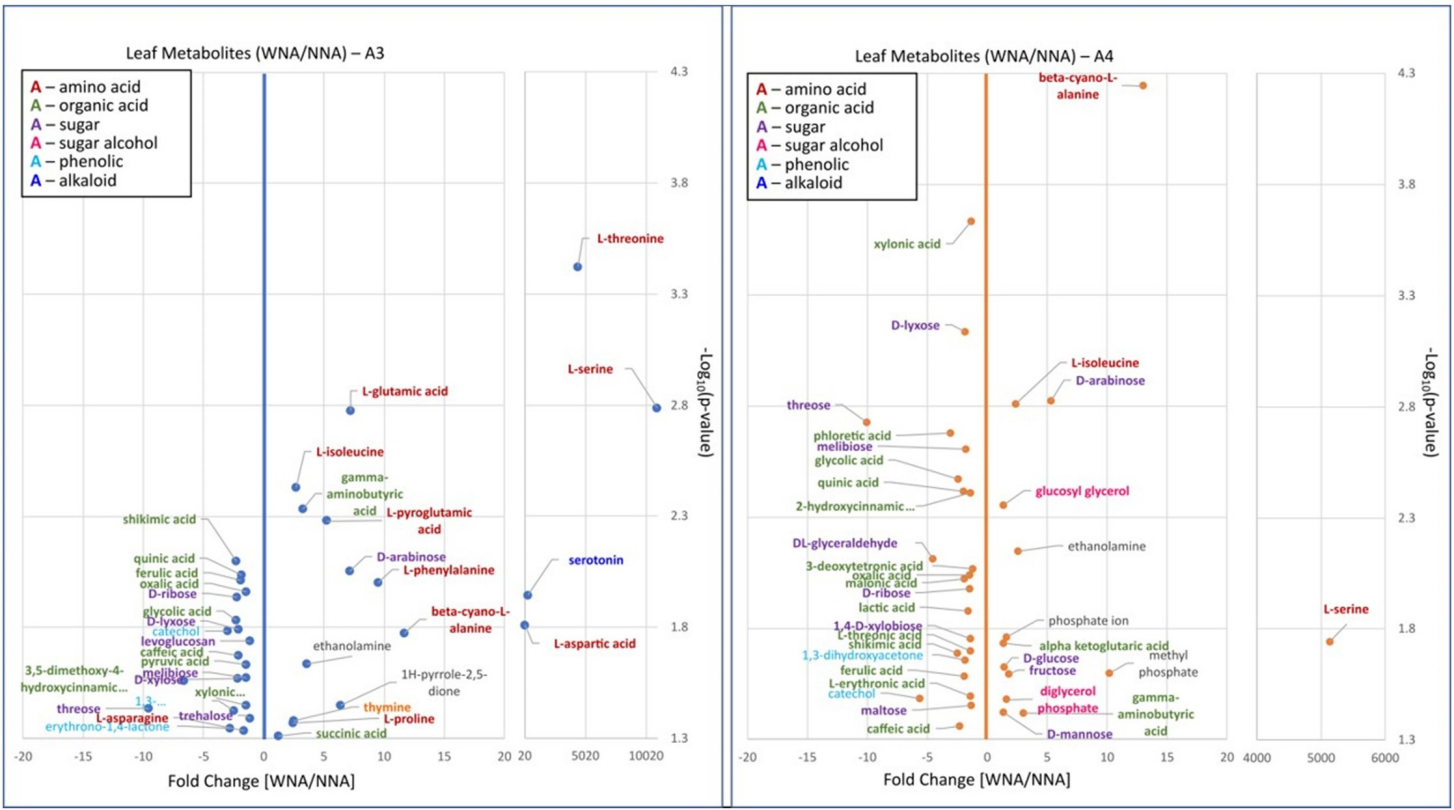
Volcano plot showing Log_10_ fold changes in leaf metabolite abundances comparing with nutrient addition (WNA) to no nutrient addition (NNA) in *Setaria italica* accessions A3 and A4.

Metabolomic analyses of leaf, root and soil samples showed a different profile of enrichment of specific classes of compounds in either the *Setaria* grown with or without nutrient addition. Fig 8 shows that the leaves and roots of plants grown in a fertilized soil (WNA) contain an enrichment of free amino acids, nucleic acids and other nitrogen containing compounds. In leaf tissue, L-serine (p <0.001), L-leucine (p <0.01), L-threonine (p <0.001), L-aspartic acid (p <0.001), L-glutamic acid (p <0.01), L-pyroglutamic acid (p <0.001), and L-phenylalanine (p <0.001) were all significantly changed with an average of at least a 5-fold increase (S1 Table) in the plants grown WNA compared to those grown without (NNA). The roots displayed significant increased abundances with a differences in enrichment of specific amino acids as compared to those identified in the leaf tissue. Here L-glutamine (p <0.05), L-asparagine (p <0.05), and L-tyrosine (p <0.01) showed at least a 5-fold increase (Table E in S1 Table) in the WNA plants compared to the NNA plants.

A further indication that *Setaria* experiencing nutrient addition were undergoing N and C balance regulation is the significant enrichment of β-cyano-L-alanine in both tissues. We observed a ~12.2-fold and 14.01-fold increases in the leaf (p <0.001) and root (p <0.001) tissue, respectively (Fig 8, Table D in S1 Table). In addition to amino acids, other nitrogen containing organic acids (e.g., gamma-aminobutyric acid, p <0.001 in leaf, p <0.01 in root), nucleic acids (e.g., thymine, p <0.001 in leaf, p <0.01 in roots; and adenosine, p <0.05 in leaf, p <0.05 in roots), alcohols (i.e., ethanolamine, p <0.001 in leaf, p <0.05 in roots), and phosphate ions (p <0.01 in leaf, p <0.05 in roots) significantly increased WNA.

**Fig 9:**
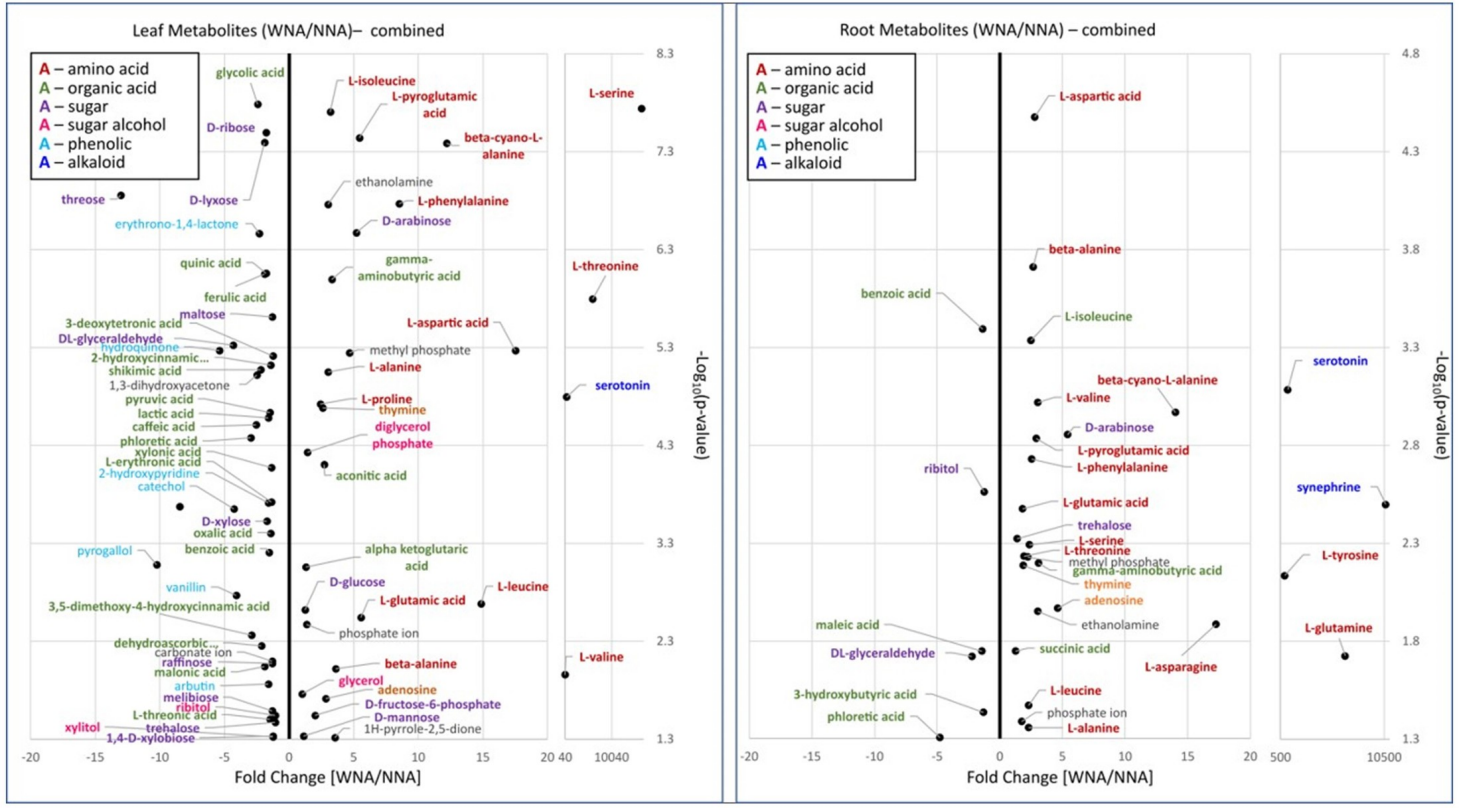
Volcano plot showing Log_10_ fold changes in leaf metabolite abundances for combined *Setaria italica* accessions grown in with nutrient addition (WNA) and no nutrient addition (NNA) soils.

In leaves especially, organic acids, and phenolics were less dominant in metabolite enrichment of WNA plants compared to NNA plants. Those phenolics with greater than 5-fold decreases (Tables D and E in S1 Table) as compared to WNA plants, included hydroquinone (p <0.001) and pyrogallol (p <0.001) in leaves and phloretic acid (p <0.05) in the roots. Phloretic acid (3-(4-hydroxyphenyl)propionic acid) showed a 4.79-fold decrease in WNA *Setaria* roots when compared to NNA roots. Of detected polyols, pyrogallol especially was found to be over 10-fold lower (Tables D and E in S1 Table) in abundance in WNA *Setaria* compared to the NNA plants. Catechol, another polyol, was also found to be significantly enriched (p <0.001) in leaf, and with a large fold decrease (i.e., 4.26 combined), especially in the leaves of A4 (p <0.05, 5.64-fold-change) as compared to A3 (p <0.05, 3.00-fold-change) (Fig 9).

While sugars, in general, were found to be significantly enriched in both the WNA and NNA plants, specific sugars were found either enriched in the WNA plants or the NNA plants with leaves producing many more significantly enriched sugars as compared to the roots. D-arabinose (P<0.001 in leaf and in roots) showed a 5.18 and 5.40-fold increase in abundance in the WNA leaves and roots, respectively. Threose (p <0.001) was found to be 13.02-fold lower in leaves of WNA plants compared to NNA plants. DL-glyceraldehyde (p <0.001 in leaves, p <0.05 in roots) showed over a 4.3-fold decrease in the leaves and 2.24-fold-decrease in roots of *Setaria* when not fertilized.

**Fig 10:**
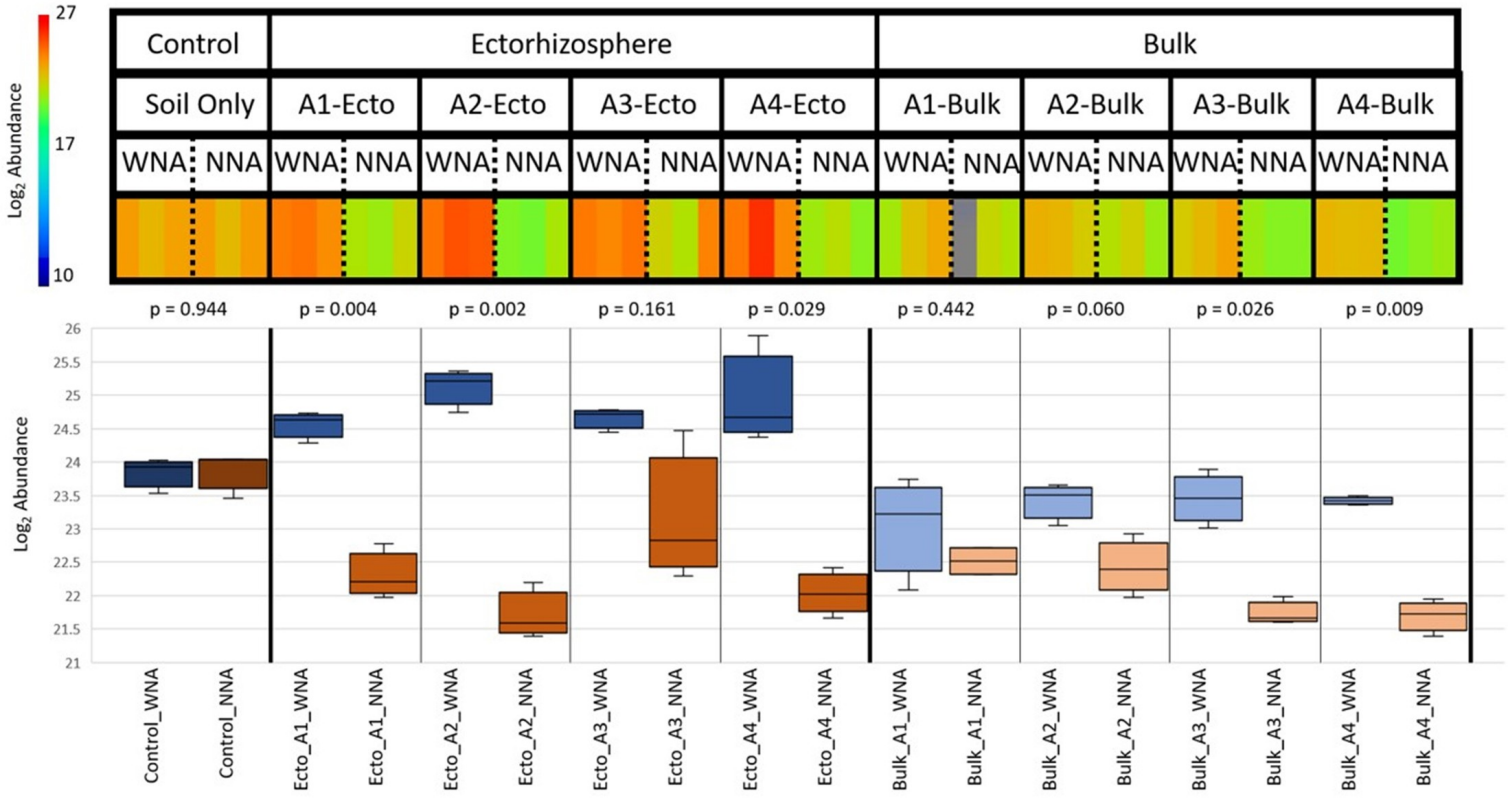
Log_2_ abundance of glycerol measured by GC-MS in the no plant control, ectorhizosphere, and bulk soil fractions for each *Setaria*. Box and whisker plot showing the Log_2_ abundance of glycerol across each accession using an ANOVA of rhizosphere metabolites (p-value < 0.01), showing significantly changed metabolites between WNA and NNA treatments.

GC-MS measurements also confirmed a difference between WNA and NNA metabolomics profiles within *Setaria* ectorhizospheres in general, and in accessions A3 and A4. A total of 64 small molecules were measured within the ectorhizosphere, with 31 of these matching to identifications within our library. WNA did not significantly alter the metabolites that were identified within bulk soil and the ectorhizosphere (S10 Fig), except for 6 metabolites: carbonate ion, fumaric acid, 3-hydroxybutyric acid, glycerol, benzoic acid, and palmitic acid. A relative decrease in carbonate ion (1.8-fold decrease, p <0.01), suggests a reduction in pH within the ectorhizosphere soil WNA. A 1.4-fold decrease (p <0.001) in the relative abundance of fumaric acid was observed as a distinguishing characteristic of the ectorhizosphere, supporting the observed relative decrease in organic acids present in leaf and root in WNA soil. In contrast, 3-hydroxybutyric acid decreased by 1.32-fold (p <0.05) in the root but increased by 1.87-fold within the ectorhizosphere. 3-hydroxybutyrate can result from branched chain amino acid catabolism (i.e., valine) during times of plant stress [43, 44]. Nutrient addition also altered the relative abundance of glycerol that increased 4.93-fold (p <0.001) when combining measurements from all *Setaria* ectorhizosphere samples and increased 8.38-fold (p <0.05) for A4 (S10 Fig). Although glycerol was measured within soil harvested from the no plant control (Fig 10), it was not measurably altered in the WNA soils (Fig 10). S7 Fig also provides evidence that the alteration of glycerol within the ectorhizosphere samples for all *Setaria* studied here is a result of plant or microbial activity, as evident when comparing its abundance measured within the surrounding bulk soil. While we did not detect glycerol in the root metabolomics analysis, the abundance of diglycerol phosphate and glycerol increased in the WNA plant leaves, which provides evidence that glycerol was produced at a higher level overall in the WNA plants.

## Discussion

While most *Setaria* studies have focused on comparing genotypic responses of the rhizosphere to various environmental conditions [8, 9, 45] or only looked at the general taxonomic structure [17], our study used TOC/TNb, 16S & ITS sequencing, FTICR-MS, and GC-MS based metabolomics to characterize the plant and ectorhizosphere response to nutrient addition in a nutrient poor soil.

To allow a multi-measurement comparison to be made on the ectorhizospheres of *Setaria*, it was crucial that the bulk and ectorhizosphere soil were successfully separated. The methods used here, and our diverse measurements, demonstrate that we were successful in performing this separation. For example, our TOC/TNb results show significantly higher values of organic C and total N in the ectorhizosphere than in the bulk soil (Fig 2), suggesting an accumulation/concentration of these nutrients by the plant-ectorhizosphere interactome. Although we did not measure metabolic activity directly, our claim is supported by previous studies. Chaparro et al. (2013) and Zhu et al. (2016) have shown that greater metabolic activity in the ectorhizosphere compared to the bulk soil occurs because nutrient addition increases root exudation into the ectorhizosphere [46, 47]. In addition, our 16S data also showed distinct separation between the ectorhizosphere and bulk soil with a large amount of overlap in OTUs between our bulk soil samples and no plant control samples (Fig 5). Similar results were shown in a study done with switchgrass [48], which further supports that our separation of the ectorhizosphere has a distinct community composition compared to bulk soil as demonstrated when comparing beta-diversity (Bray-Curtis) (S11 Fig).

The use of FTICR-MS to distinguish the ectorhizosphere from bulk soil represents a more recent application of this capability. Previous applications of FTICR-MS in soils have been used to characterize soil organic matter [28], dissolved organic matter [49], and determine how the rhizosphere can absorb metal pollutants [50]. By using FTICR-MS here, we not only identified separate soil fractions based upon organic chemical composition (S3 Fig), but also demonstrated that alterations to SOM chemical classes differs with *Setaria spp*. and within different accessions of the same species, as reported here for accessions A3 and A4. For example, under NNA the protein and amino sugar chemical classes could serve as N sources for A3, while for A4, a lower demand for N could be hypothesized based upon less alteration of these chemical classes. WNA, lignin decreases substantially in A3, yet not significantly for A4, while the opposite was observed for the tannin chemical class (Fig 4). The alteration in these latter chemical classes leads us to hypothesize that this outcome is a result of differences in rhizosphere priming [51]. Dijkstra et al. (2013), defined rhizosphere priming as a change in the composition of SOM caused by root activity, which can be affected by nutrient availability [51]. If correct, our observations further suggest that differences in rhizosphere priming are a function of plant genotype (here accession, as the genotype of each *Setaria* was not investigated), with alterations in different chemical classes brought about by the interaction of the plant and its rhizosphere commensals; differences in root exudation for regulating these SOM chemical classes as sources and sinks of C and N. Further experimentation is needed to validate this hypothesis.

The comparison of metabolites identified in our ‘no plant’ controls to those identified in the ectorhizosphere, bulk soils, leaves and roots provided examples of the enrichment of specific metabolites, which accumulated in either the roots, leaves and in the ectorhizosphere during nutrient deplete or replete conditions. Enrichment of specific metabolites in the roots could be exudated, which in turn could be used to add positive or negative growth pressures on specific ectorhizosphere microbes. For example, glutamic acid (Glu) and aspartic acid (Asp) where enriched in the leaves but the amine-enriched relatives to Glu and Asp, glutamine (Gln) and asparagine (Asn) were highly enriched in the roots. This might be indicative of the processes *Setaria* employ to balance source-sink relationships of C and N. Differential accumulation of distinct amino acids in distinct plant tissues supports the idea that amino acids play a central role in N incorporation after uptake from soil, along with the translocation, utilization, and metabolism within plants. All NH_4_^+^ derived from soil or produced from NO_3_^-^ reduction is first channeled through the glutamine synthetase (GS) reaction [52]. GS catalyzes the fixation of NH_4_^+^ into Glu to form glutamic acid. Glutamine and glutamic acid then can be utilized as amino group donors as well as N transport molecules [53]. GS activity has been tied to metabolic and environmental changes and has been linked to the balance of C and N metabolism [54–56] where Glu levels act as a nutritional status sensor for plants. Further interconversions from Glu to Gln and to other amino acids, especially Asn, are then possible.

Source-sink differences for C and N observed with *Setaria* could also come about through our observation of large fold increases of beta-cyano-L-alanine coupled with high abundances of L-phenylalanine (Phe) in the leaves and L-tyrosine (Tyr) in the roots (Figs 8 and 9). This observation suggests that WNA *Setaria* is utilizing the cyanoamino acid pathway to produce hydrogen cyanide (HCN) to act as a negative regulator of nitrate reductase (NR) [57]. NR controls nitrate to nitrite conversions enabling the downstream conversion of nitrite to hydroxylamine to ammonium in the plant cells. The ammonium is then coupled with carbon skeletons (i.e., 3-phosphoglycerate) produced in photosynthesis to produce amino acids. If production of ammonium ions is not stoichiometrically coupled with carbon skeleton production, then a toxic buildup of nitrate, hydroxylamine and ammonium can occur. This regulation however also needs to be controlled as HCN is also toxic to the plant in large enough quantities. To accomplish cyanide detoxification, higher plants convert the cyanide into beta-cyano-L-alanine, which is further converted enzymatically by two different pathways to either L-asparagine, which we found to be highly abundant in the WNA roots, or L-aspartate [58], which we found to be highly abundant in the WNA leaves (Fig 8).

The alkaloid synephrine may also play an important role in our observed nutrient acquisition differences in *Setaria*. We detected a large increase in synephrine in the WNA plants compared to the NNA plants. We hypothesize that this alkaloid may be an important response metabolite in nutrient-rich environments. Generally, synephrine research has focused on its use as a vasoconstrictor agent in animals and humans as a treatment to shock and sometimes asthma [59, 60]. The limited research on synephrine in plant-soil interactions shows that Anthrobacter which is an Actinobacteria, can use synephrine as a sole carbon and nitrogen source [61]. Future studies should investigate further synephrine’s role in nitrogen utilization or possible microbiome remodeling in *Setaria*.

A high abundance of glycerol observed in the ectorhizosphere WNA of all *Setaria* grown in this study indicates nutrient addition enhances glycerol uptake in a nutrient poor soil. Glycerol has been detected in other plant studies as a root exudate [46, 62]. Miller et al. (2019) detected glycerol in a non-targeted metabolomics analysis of sorghum rhizospheres, in sand, clay or soil media [63] where they observed much higher levels of glycerol in their no-plant control compared to the soil growing sorghum. In our soil analysis here, we also had no-plant controls which did show glycerol levels to be higher when compared to the potted plants having NNA, but lower in the ectorhizosphere of plants WNA (S10 Fig). We observed that glycerol is significantly greater in the ectorhizosphere soil for both A3 and A4 while the bulk soil shows a much smaller change. This suggests that *Setaria* might utilize glycerol in a nutrient poor environment to mitigate stress. Miller et al. (2019) suggested that glycerol may act as both a plant root exudate and a rhizosphere-abated metabolite [63]. In this study, glycerol was considered an osmotic stress protectant for both plants and microorganisms, and it has been documented that glycerol can be used by bacteria such as *Pseudomonas putida*, a soil monoderm and Actinobacteria, as a sole carbon source [64]. It also has been shown to possess auxin-like activity, negatively affecting root growth [65]. In this study, exogenous glycerol applied to *Arabidopsis* inhibited primary root growth and altered lateral root development. This phenotype appeared concurrently with increased endogenous glycerol 3-phosphate (G3P), H_2_O_2_ and decreased phosphate levels in roots. In plants with exogenously applied glycerol, free auxin content increased by 46%, suggesting that glycerol likely altered normal auxin distribution thus affecting root development. It is likely that the presence of glycerol in the plant and as a root exudate, is tied to nutrient availability and when *Setaria* is grown under nutrient limited conditions, the plant exudate composition might enable enrichment of microorganisms which favor glycerol as a carbon source. This alteration in the level of glycerol then might act to alter auxin levels and thus root architecture which are better suited to low nutrient soils (e.g., high surface area fine root formation).

Other compounds found in the soil metabolite analysis revealed enrichments of benzoic and palmitic acid. Benzoic acid and palmitic acid are organic acids which have the capability of altering soil microbiomes. We found both compounds to be significantly enriched in the loose bound soil surrounding the roots of setaria grown WNA as compared to setaria grown NNA (S10 Fig). In prior work [66], benzoic acid added to soil with peanut plants have been shown to be quickly consumed and metabolized by bacteria such as Burkholderia, whereas AD3 and actinobacteria have been shown to be reduced in benzoic acid treated soils. In watermelon studies, palmitic acid addition to the soil, decreased the severity of Fusarium wilt, changed the bacteria microbiome composition, and overall promoted the growth of watermelon [67]. It has therefore been hypothesized that plants exude such organic acids as a mechanism to remodel the soil bacteria for species which metabolize the acids making plants less susceptible to fungal pathogens [68]. Our data suggest that Setaria with nutrient amendment might be exuding such organic acids to the soil as a mechanism to inhibit pathogenic fungi.

In conclusion, our comparison of A3 and A4suggests that A3 responds better to nutrient amendment, while A4 is better adapted to nutrient poor soil conditions. While a greater analysis of metabolomics data (and SOM and community structure) did not extend to A1 and A2, measured global responses of these accessions showed similarity to A3. And, it is possible that for these accessions hydroquinone and serotonin (a tyrosine derived alkaloid like synephrine) may be responsible for this finding. Hydroquinone is known as a growth stimulator in small concentrations and as a growth inhibitor at high concentrations [69]. Hydroquinone might act as a growth inhibitor for some *Setaria spp* or accessions (here, accession A3) grown with limited nutrient availability. For example, growth indicators for A3 (above and below ground biomass, plant height; S12 Fig) differed to a larger degree than for A4, supporting the observed enrichment of hydroquinone under NNA relative to WNA. However, WNA above ground growth may be promoted via serotonin, which was observed to be greater in the leaves of A3 WNA, but not measured for A4 (Fig 8). Admittedly, the overall role of serotonin in plants is not well known, but it has been suggested to play a role in plant growth and root architecture [70], along with glycerol. While not undertaken here, future studies directly measuring which metabolites are actively exuded to the rhizosphere using stable isotope tracing would be an informative addition to this study as well as experiments to characterize what specific influence the alkaloids serotonin and synephrine have on soil microbiome dynamics, if any, would be interesting to pursue. A future genome-wide association experiment (GWAS) could also further elucidate why genetically, A3 responded differently than A4 with and without nutrient addition.

## Supporting information

S1 Fig Workflow including sample amounts and normalization broken down by individual analysis. (TIF)

S2 Fig Average dry weight of *Setaria* total biomass with nutrient addition (WNA) and no nutrient addition (NNA). (TIF)

S3 Fig Non-metric multidimensional scaling of FTICR-MS measured soil organic matter profiles based on soil fraction. (TIF)

S4 Fig Alpha diversity measure of 16S bacterial diversity shows that there are no significant differences in bacterial community between treatments within each accession (paired t-test). (TIF)

S5 Fig The relative abundance of bacterial OTUs across all treatments shown at the phyla level. (TIF)

S6 Fig 4-way Venn Diagram showing the unique 16S OTUs between *Setaria italica* accessions A3 and A4 based on treatment. (TIF)

S7 Fig 4-way Venn Diagram showing the unique ITS OTUs between *Setaria italica* accessions A3 and A4 based on treatment. (TIF)

S8 Fig Fold-change of log_2_ abundances, measured for fungal OTUs assigned to phyla, comparing no nutrient addition (NNA) between *Setaria italica* accessions A3 and A4 in the ectorhizosphere. (TIF)

S9 Fig Fold-change of log_2_ abundances, measured for fungal OTUs assigned to phyla, comparing with nutrient addition (WNA) between *Setaria italica* accessions A3 and A4 in the ectorhizosphere. (TIF)

S10 Fig Volcano plot showing Log_10_ fold changes in rhizosphere and loose bound soil metabolites for combined *Setaria italica* accessions as well as *Setaria italica* accessions A3 and A4 grown with nutrient addition (WNA) and no nutrient addition (NNA) soils. (TIF)

S11 Fig Beta Diversity of 16S bacterial data using Bray-Curtis dissimilarity and an analysis of similarity (ANOSIM) to determine the difference of the bacterial community between each accession and treatment (ANOSIM, R = 0.57, p = 0.0001). (TIF)

S12 Fig *Setaria viridis* (A1), and *Setaria italica* accessions A2–A4 grown with nutrient addition (Left) and no nutrient addition (Right). Vertical scale indicates 1 cm.
(TIF)

S1 Table Contains supporting Tables A through E.
(XLSX)

## References

[1] Rominger JM. Taxonomy of Setaria (Gramineae) in North America. Urbana-Champaign: Urbana, University of Illinois Press; 1962.

[2] Li P, Brutnell TP. *Setaria viridis* and *Setaria italica*, model genetic systems for the Panicoid grasses. J Exp Bot. 2011;62(9):3031–7. doi: 10.1093/jxb/err096 21459768

[3] Sanaullah M, Blagodatskaya E, Chabbi A, Rumpel C, Kuzyakov Y. Drought effects on microbial biomass and enzyme activities in the rhizosphere of grasses depend on plant community composition. Appl Soil Ecol. 2011;48(1):38–44.

[4] Hu H, Mauro-Herrera M, Doust AN. Domestication and improvement in the model C4 grass, Setaria. Front Plant Sci. 2018;9. doi: 10.3389/fpls.2018.00009 29896214 PMC5986938

[5] Jayaraman A, Puranik S, Rai NK, Vidapu S, Sahu PP, Lata C, et al. cDNA-AFLP analysis reveals differential gene expression in response to salt stress in Foxtail Millet (*Setaria italica* L.). Mol Biotechnol. 2008;40(3):241–51. doi: 10.1007/s12033-008-9081-4 18592419

[6] Jia GQ, Huang XH, Zhi H, Zhao Y, Zhao Q, Li WJ, et al. A haplotype map of genomic variations and genome-wide association studies of agronomic traits in foxtail millet *(Setaria italica*). Nat Genet. 2013;45(8):957–U167. doi: 10.1038/ng.2673 23793027

[7] Jin T, Wang YY, Huang YY, Xu J, Zhang PF, Wang N, et al. Taxonomic structure and functional association of foxtail millet root microbiome. Gigascience. 2017;6(10). doi: 10.1093/gigascience/gix089 29050374 PMC7059795

[8] Lata C, Bhutty S, Bahadur RP, Majee M, Prasad M. Association of an SNP in a novel DREB2-like gene SiDREB2 with stress tolerance in foxtail millet [*Setaria italica* (L.)]. Journal of Experimental Botany. 2011;62(10):3387–401. doi: 10.1093/jxb/err016 21414959 PMC3130168

[9] Lata C, Gupta S, Prasad M. Foxtail millet: a model crop for genetic and genomic studies in bioenergy grasses. Crit Rev Biotechnol. 2013;33(3):328–43. doi: 10.3109/07388551.2012.716809 22985089

[10] Pan JW, Li Z, Wang QG, Garrell AK, Liu M, Guan YA, et al. Comparative proteomic investigation of drought responses in foxtail millet. Bmc Plant Biol. 2018;18. doi: 10.1186/s12870-018-1232-6 30497407 PMC6267058

[11] Saha P, Sade N, Arzani A, Wilhelmi MDR, Coe KM, Li BS, et al. Effects of abiotic stress on physiological plasticity and water use of *Setaria viridis* (L.). Plant Sci. 2016;251:128–38. doi: 10.1016/j.plantsci.2016.06.011 27593471

[12] Veeranagamallaiah G, Jyothsnakumari G, Thippeswamy M, Reddy PCO, Surabhi GK, Sriranganayakulu G, et al. Proteomic analysis of salt stress responses in foxtail millet (*Setaria italica* L. cv. *Prasad*) seedlings. Plant Sci. 2008;175(5):631–41.

[13] Gu S. Relationship between foxtail millet growth and environmental factors. In: Gu S, editor. Foxtail millet cultivation and production in China. Beijing, China: Chinese Agricultural Press; 1987. p. 63–71.

[14] Brutnell TP, Wang L, Swartwood K, Goldschmidt A, Jackson D, Zhu XG, et al. *Setaria viridis*: a model for C-4 photosynthesis. Plant Cell. 2010;22(8):2537–44. doi: 10.1105/tpc.110.075309 20693355 PMC2947182

[15] Zhang GY, Liu X, Quan ZW, Cheng SF, Xu X, Pan SK, et al. Genome sequence of foxtail millet (*Setaria italica*) provides insights into grass evolution and biofuel potential. Nat Biotechnol. 2012;30(6):549–+. doi: 10.1038/nbt.2195 22580950

[16] McNear Jr. DH. The rhizosphere—roots, soil and everything in between. Nature Education Knowledge. 2013;4(3):1.

[17] Okon Y, Heytler PG, Hardy RWF. N2-Fixation by Azospirillum-Brasilense and its incorporation into host Setaria-Italica. Appl Environ Microb. 1983;46(3):694–7. doi: 10.1128/aem.46.3.694-697.1983 16346386 PMC239336

[18] Nadeem F, Ahmad Z, Wang RF, Han JN, Shen Q, Chang FR, et al. Foxtail Millet [*Setaria italica* (L.) *Beauv*.] grown under low nitrogen shows a smaller root system, enhanced biomass accumulation, and nitrate transporter expression. Front Plant Sci. 2018;9. doi: 10.3389/fpls.2018.00009 29520286 PMC5826958

[19] Kawasaki A, Donn S, Ryan PR, Mathesius U, Devilla R, Jones A, et al. Microbiome and exudates of the root and rhizosphere of *Brachypodium distachyon*, a model for wheat. Plos One. 2016;11(10). 10.1371/journal.pone.0164533 PMC5058512 27727301

[20] Caporaso JG, Lauber CL, Walters WA, Berg-Lyons D, Huntley J, Fierer N, et al. Ultra-high-throughput microbial community analysis on the Illumina HiSeq and MiSeq platforms. Isme J. 2012;6(8):1621–4. doi: 10.1038/ismej.2012.8 22402401 PMC3400413

[21] Brown J, Zavoshy N, Brislawn CJ, McCue LA. Hundo: a Snakemake workflow for microbial community sequence data. Peerj Preprints. 2018;6:e27272v1.

[22] Quast C, Pruesse E, Yilmaz P, Gerken J, Schweer T, Yarza P, et al. The SILVA ribosomal RNA gene database project: improved data processing and web-based tools. Nucleic Acids Res. 2013;41(D1):D590–D6. doi: 10.1093/nar/gks1219 23193283 PMC3531112

[23] Yilmaz P, Parfrey LW, Yarza P, Gerken J, Pruesse E, Quast C, et al. The SILVA and "All-species Living Tree Project (LTP)" taxonomic frameworks. Nucleic Acids Res. 2014;42(D1):D643–D8. doi: 10.1093/nar/gkt1209 24293649 PMC3965112

[24] Koljalg U, Nilsson HR, Schigel D, Tedersoo L, Larsson KH, May TW, et al. The taxonhypothesis paradigm-on the unambiguous detection and communication of taxa. microorganisms. 2020;8(12). doi: 10.3390/microorganisms8121910 33266327 PMC7760934

[25] Nilsson RH, Larsson KH, Taylor AFS, Bengtsson-Palme J, Jeppesen TS, Schigel D, et al. The UNITE database for molecular identification of fungi: handling dark taxa and parallel taxonomic classifications. Nucleic Acids Res. 2019;47(D1):D259–D64. doi: 10.1093/nar/gky1022 30371820 PMC6324048

[26] Altschul SF, Gish W, Miller W, Myers EW, Lipman DJ. Basic local alignment search tool. J Mol Biol. 1990;215(3):403–10. doi: 10.1016/S0022-2836(05)80360-2 2231712

[27] Stratton KG, Webb-Robertson BJM, McCue LA, Stanfill B, Claborne D, Godinez I, et al. pmartR: quality control and statistics for mass spectrometry-based biological data. J Proteome Res. 2019;18(3):1418–25. doi: 10.1021/acs.jproteome.8b00760 30638385 PMC6750869

[28] Tfaily MM, Chu RK, Toyoda J, Tolic N, Robinson EW, Pasa-Tolic L, et al. Sequential extraction protocol for organic matter from soils and sediments using high resolution mass spectrometry. Anal Chim Acta. 2017;972:54–61. doi: 10.1016/j.aca.2017.03.031 28495096

[29] Dittmar T, Koch B, Hertkorn N, Kattner G. A simple and efficient method for the solid-phase extraction of dissolved organic matter (SPE-DOM) from seawater. Limnol Oceanogr-Meth. 2008;6:230–5.

[30] Tolic N, Liu Y, Liyu A, Shen YF, Tfaily MM, Kujawinski EB, et al. Formularity: software for automated formula assignment of natural and other organic matter from ultrahigh-resolution mass spectra. Anal Chem. 2017;89(23):12659–65. doi: 10.1021/acs.analchem.7b03318 29120613

[31] Kujawinski EB, Behn MD. Automated analysis of electrospray ionization Fourier transform ion cyclotron resonance mass spectra of natural organic matter. Anal Chem. 2006;78(13):4363–73. doi: 10.1021/ac0600306 16808443

[32] Minor EC, Steinbring CJ, Longnecker K, Kujawinski EB. Characterization of dissolved organic matter in Lake Superior and its watershed using ultrahigh resolution mass spectrometry. Org Geochem. 2012;43:1–11.

[33] Kim S, Kramer RW, Hatcher PG. Graphical method for analysis of ultrahigh-resolution broadband mass spectra of natural organic matter, the van Krevelen diagram. Anal Chem. 2003;75(20):5336–44. doi: 10.1021/ac034415p 14710810

[34] Wu ZG, Rodgers RP, Marshall AG. Two- and three-dimensional van Krevelen diagrams: A graphical analysis complementary to the Kendrick mass plot for sorting elemental compositions of complex organic mixtures based on ultrahigh-resolution broadband Fourier transform ion cyclotron resonance mass measurements. Anal Chem. 2004;76(9):2511–6. doi: 10.1021/ac0355449 15117191

[35] Folch J, Ascoli I, Lees M, Meath JA, Lebaron FN. Preparation of lipide extracts from brain tissue. J Biol Chem. 1951;191(2):833–41. 14861228

[36] Nakayasu ES, Nicora CD, Sims AC, Burnum-Johnson KE, Kim YM, Kyle JE, et al. MPLEx: a robust and universal protocol for single-sample integrative proteomic, metabolomic, and lipidomic analyses. Msystems. 2016;1(3). 10.1128/mSystems.00043-16 PMC5069757 27822525

[37] Kim YM, Nowack S, Olsen MT, Becraft ED, Wood JM, Thiel V, et al. Diel metabolomics analysis of a hot spring chlorophototrophic microbial mat leads to new hypotheses of community member metabolisms. Front Microbiol. 2015;6. doi: 10.3389/fmicb.2015.00006 25941514 PMC4400912

[38] Hiller K, Hangebrauk J, Jager C, Spura J, Schreiber K, Schomburg D. MetaboliteDetector: comprehensive analysis tool for targeted and nontargeted GC/MS based metabolome analysis. Anal Chem. 2009;81(9):3429–39. doi: 10.1021/ac802689c 19358599

[39] Polpitiya AD, Qian WJ, Jaitly N, Petyuk VA, Adkins JN, Camp DG, et al. DAnTE: a statistical tool for quantitative analysis of -omics data. Bioinformatics. 2008;24(13):1556–8. doi: 10.1093/bioinformatics/btn217 18453552 PMC2692489

[40] Dhawi F, Datta R, Ramakrishna W. Metabolomics, biomass and lignocellulosic total sugars analysis in foxtain millet (*Setaria italica*) inoculated with different combinations of plant growth promoting bacteria and mycorrhiza. Communications in Plant Sciences. 2018;8:8–14.

[41] Anandan R, Dharumadurai D, Manogaran GP. An introduction to Actinobacteria. IntechOpen. 2016;DOI:105772/62329.

[42] Tang ZX, Sun XL, Luo ZK, He NP, Sun OJ. Effects of temperature, soil substrate, and microbial community on carbon mineralization across three climatically contrasting forest sites. Ecol Evol. 2018;8(2):879–91. doi: 10.1002/ece3.3708 29375762 PMC5773329

[43] Schertl P, Danne L, Braun HP. 3-Hydroxyisobutyrate dehydrogenase is involved in both, Valine and Isoleucine degradation in *Arabidopsis thaliana*. Plant Physiol. 2017;175(1):51–61. doi: 10.1104/pp.17.00649 28705827 PMC5580760

[44] Hildebrandt TM, Nesi AN, Araujo WL, Braun HP. Amino acid catabolism in plants. Mol Plant. 2015;8(11):1563–79. doi: 10.1016/j.molp.2015.09.005 26384576

[45] Ceasar SA, Ramakrishnan M, Vinod KK, Roch GV, Upadhyaya HD, Baker A, et al. Phenotypic responses of foxtail millet (*Setaria italica*) genotypes to phosphate supply under greenhouse and natural field conditions. Plos One. 2020;15(6). doi: 10.1371/journal.pone.0233896 32492057 PMC7269269

[46] Chaparro JM, Badri DV, Bakker MG, Sugiyama A, Manter DK, Vivanco JM. Root exudation of phytochemicals in Arabidopsis follows specific patterns that are developmentally programmed and correlate with soil microbial functions. Plos One. 2013;8(2). doi: 10.1371/journal.pone.0055731 23383346 PMC3562227

[47] Zhu SS, Vivanco JM, Manter DK. Nitrogen fertilizer rate affects root exudation, the rhizosphere microbiome and nitrogen-use-efficiency of maize. Appl Soil Ecol. 2016;107:324–33.

[48] Chaudhary DR, Saxena J, Lorenz N, Dick RP. Distribution of recently fixed photosynthate in a switchgrass plant-soil system. Plant Soil Environ. 2012;58(6):249–55.

[49] Xu W, Gao A, He C, Shi Q, Hou Z, Zhao HZ. Using ESI FT-ICR MS to characterize dissolved organic matter in salt lakes with different salinity. Environ Sci Technol. 2020;54(20):12929–37. doi: 10.1021/acs.est.0c01681 33040523

[50] Wen JJ, Li ZW, Luo NL, Huang M, Yang R, Zeng GM. Investigating organic matter properties affecting the binding behavior of heavy metals in the rhizosphere of wetlands. Ecotox Environ Safe. 2018;162:184–91. doi: 10.1016/j.ecoenv.2018.06.083 29990730

[51] Dijkstra FA, Carrillo Y, Pendall E, Morgan JA. Rhizosphere priming: a nutrient perspective. Front Microbiol. 2013;4. doi: 10.3389/fmicb.2013.00004 23908649 PMC3725428

[52] Prinsi B, Espen L. Mineral nitrogen sources differently affect root glutamine synthetase isoforms and amino acid balance among organs in maize. Bmc Plant Biol. 2015;15. doi: 10.1186/s12870-014-0394-0 25886826 PMC4393875

[53] Hirel B, Lea PJ. Ammonia Assimilation. In: Lea PJ, Morot-Gaudry JF, editors. Berlin, Heidelberg: Springer Berlin Heidelberg; 2001.

[54] Forde BG, Lea PJ. Glutamate in plants: metabolism, regulation, and signalling. Journal of Experimental Botany. 2007;58(9):2339–58. doi: 10.1093/jxb/erm121 17578865

[55] Lea PJ, Azevedo RA. Nitrogen use efficience, 2. Amin acid metabolism. Annals of Applied Biology. 2007;151:269–75.

[56] Thomsen HC, Eriksson D, Moller IS, Schjoerring JK. Cytosolic glutamine synthetase: a target for improvement of crop nitrogen use efficiency? Trends Plant Sci. 2014;19(10):656–63. doi: 10.1016/j.tplants.2014.06.002 25017701

[57] Siegien I, Bogatek R. Cyanide action in plants—from toxic to regulatory. Acta Physiol Plant. 2006;28(5):483–97.

[58] Castric PA, Conn EE, Farnden KJF. Cyanide metabolism in higher-plants .5. Formation of Asparagine from Beta-Cyanoalanine. Arch Biochem Biophys. 1972;152(1):62–&. doi: 10.1016/0003-9861(72)90193-2 4627358

[59] Stohs SJ, Preuss HG, Shara M. The safety of *Citrus aurantium* (Bitter Orange) and its primary protoalkaloid p-Synephrine. Phytother Res. 2011;25(10):1421–8. doi: 10.1002/ptr.3490 21480414

[60] Wu QC, Li RS, Soromou LW, Chen N, Yuan X, Sun GQ, et al. p-Synephrine suppresses lipopolysaccharide-induced acute lung injury by inhibition of the NF-kappa B signaling pathway. Inflamm Res. 2014;63(6):429–39. doi: 10.1007/s00011-014-0715-7 24487736

[61] Devi NA, Kutty RK, Vasantharajan VN, Rao PVS. Microbial metabolism of phenolic amines—degradation of Dl-Synephrine by an unidentified Arthrobacter. J Bacteriol. 1975;122(3):866–73. doi: 10.1128/jb.122.3.866-873.1975 1150621 PMC246136

[62] Neumann G, Bott S, Ohler MA, Mock HP, Lippmann R, Grosch R, et al. Root exudation and root development of lettuce (*Lactuca sativa* L. cv. *Tizian*) as affected by different soils. Front Microbiol. 2014;5. doi: 10.3389/fmicb.2014.00005 24478764 PMC3901204

[63] Miller SB, Heuberger AL, Broeckling CD, Jahn CE. Non-targeted metabolomics reveals Sorghum rhizosphere-associated exudates are influenced by the belowground interaction of substrate and Sorghum genotype. Int J Mol Sci. 2019;20(2). 10.3390/ijms20020431 PMC6358735 30669498

[64] Nikel PI, Romero-Campero FJ, Zeidman JA, Goni-Moreno A, de Lorenzo V. The Glycerol-dependent metabolic persistence of *Pseudomonas putida* KT2440 reflects the regulatory logic of the GlpR repressor. Mbio. 2015;6(2). doi: 10.1128/mBio.00340-15 25827416 PMC4453509

[65] Hu J, Zhang YH, Wang JF, Zhou YM. Glycerol affects root development through regulation of multiple pathways in Arabidopsis. Plos One. 2014;9(1). doi: 10.1371/journal.pone.0086269 24465999 PMC3899222

[66] Liu JG, Li XG, Jia ZJ, Zhang TL, Wang XX. Effect of benzoic acid on soil microbial communities associated with soilborne peanut diseases. Appl Soil Ecol. 2017;110:34–42.

[67] Ma KX, Kou JM, Rahman MKU, Du WT, Liang XY, Wu FZ, et al. Palmitic acid mediated change of rhizosphere and alleviation of Fusarium wilt disease in watermelon. Saudi J Biol Sci. 2021;28(6):3616–23. doi: 10.1016/j.sjbs.2021.03.040 34121905 PMC8176049

[68] Liu JG, Wang XX, Zhang TL, Li XG. Assessment of active bacteria metabolizing phenolic acids in the peanut (*Arachis hypogaea* L.) rhizosphere. Microbiol Res. 2017;205:118–24. doi: 10.1016/j.micres.2017.09.005 28942837

[69] Kamran M, Khan AL, Ali L, Hussain J, Waqas M, Al-Harrasi A, et al. Hydroquinone; a novel bioactive compound from plant-derived smoke can cue ceed germination of lettuce. Front Chem. 2017;5. doi: 10.3389/fchem.2017.00005 28553632 PMC5427145

[70] Ramakrishna A, Giridhar P, Ravishankar GA. Phytoserotonin: a review. Plant Signal Behav. 2011;6(6):800–9. doi: 10.4161/psb.6.6.15242 21617371 PMC3218476

