# Supplementary figures and images for: Deciphering the microbial and molecular responses of geographically diverse Setaria accessions grown in a nutrient-poor soil
Source: PLoS One. 2021 Dec 8;16(12):e0259937. doi: 10.1371/journal.pone.0259937 (PMC8654227; doi:10.1371/journal.pone.0259937)

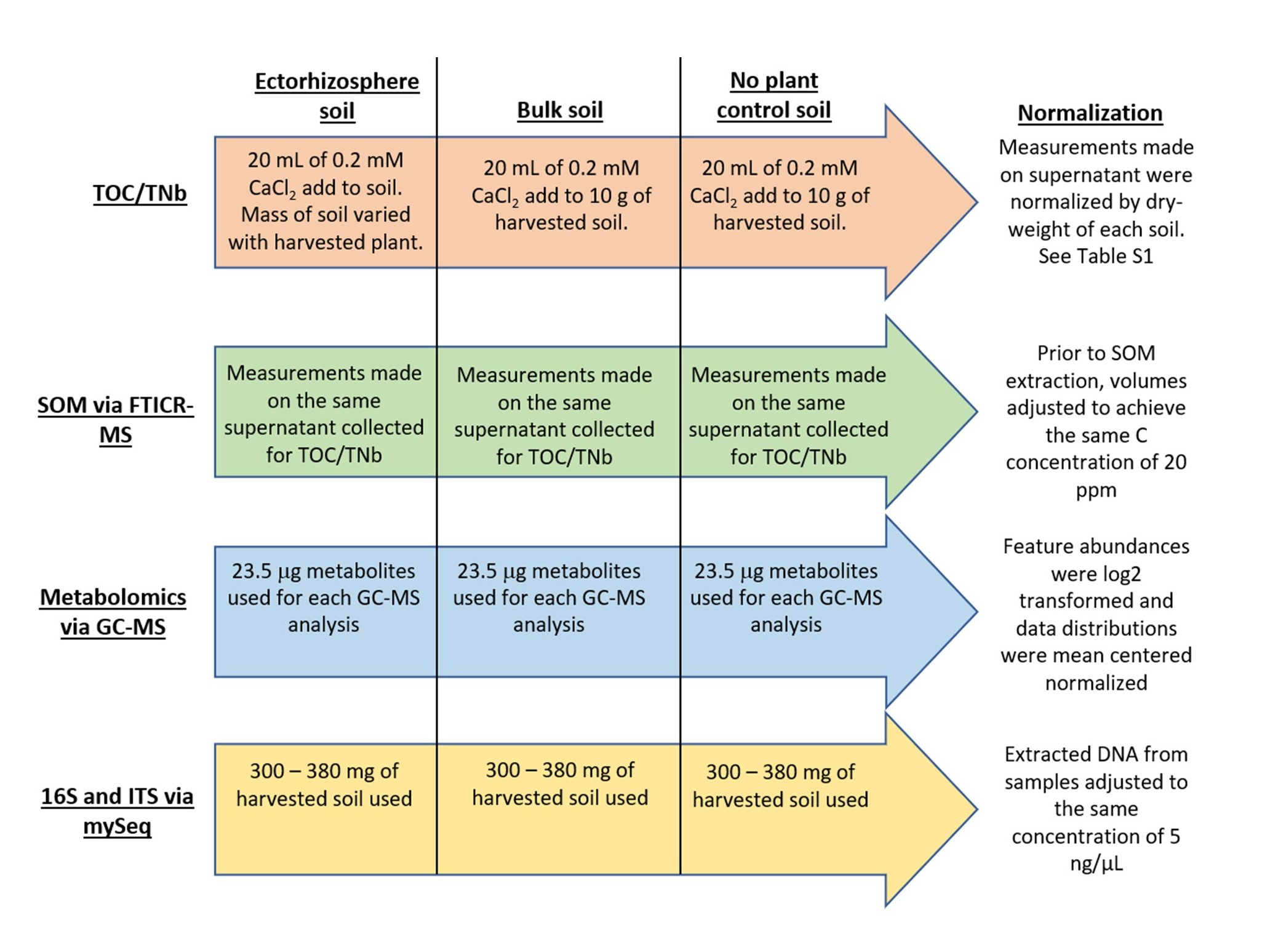

Supplement: S1 Fig — (TIF) [file pone.0259937.s001.tif]

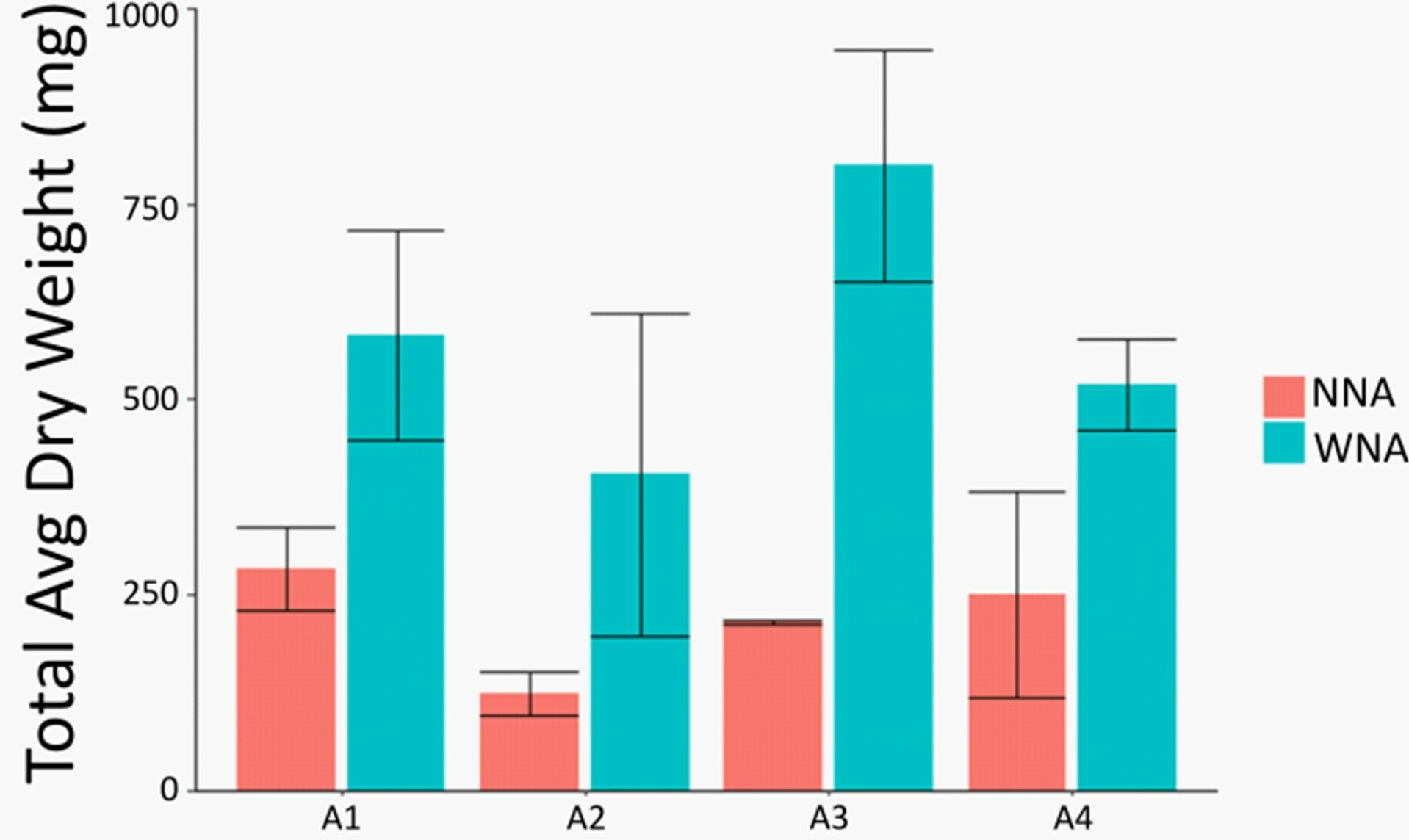

Supplement: S2 Fig — (TIF) [file pone.0259937.s002.tif]

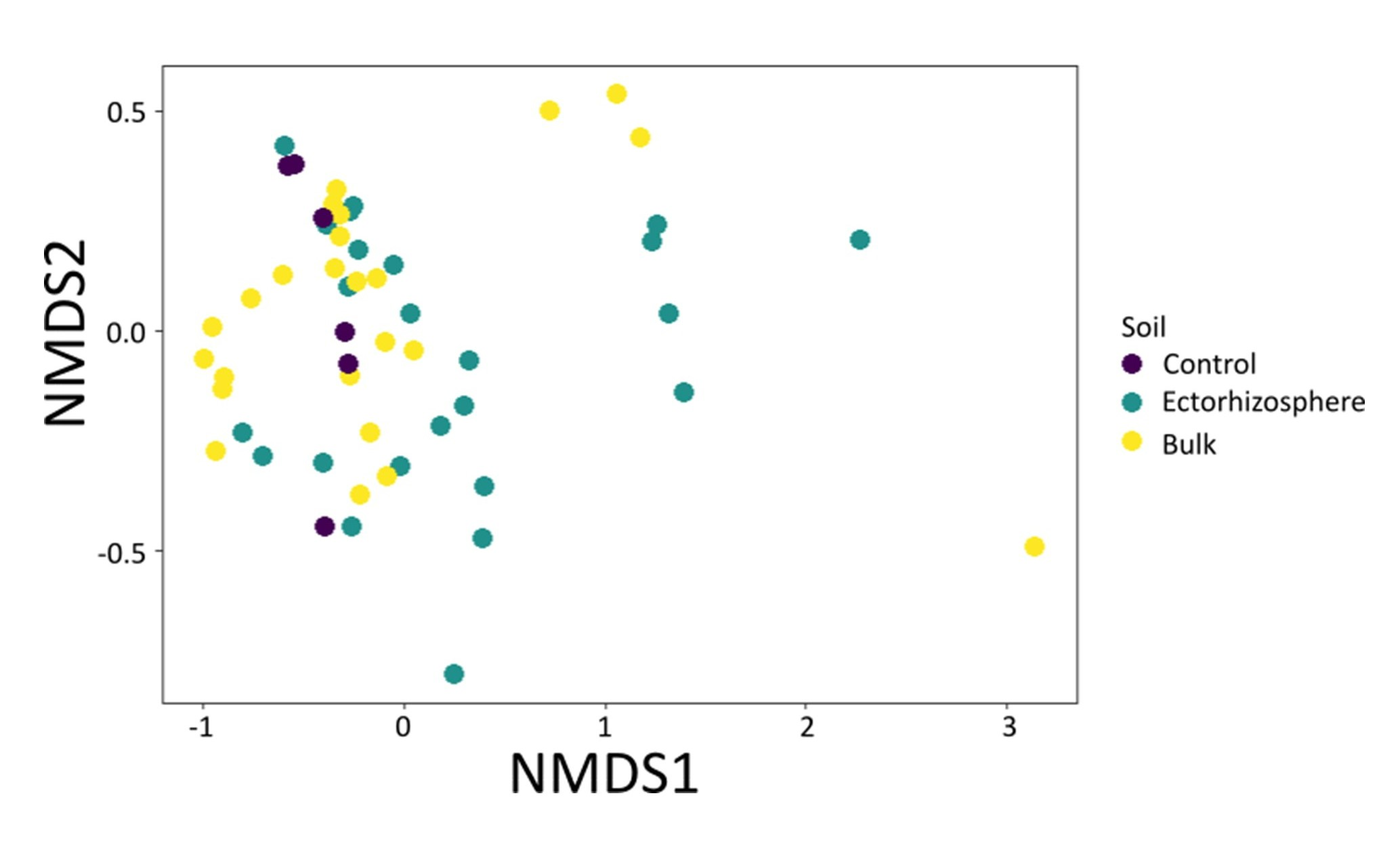

Supplement: S3 Fig — (TIF) [file pone.0259937.s003.tif]

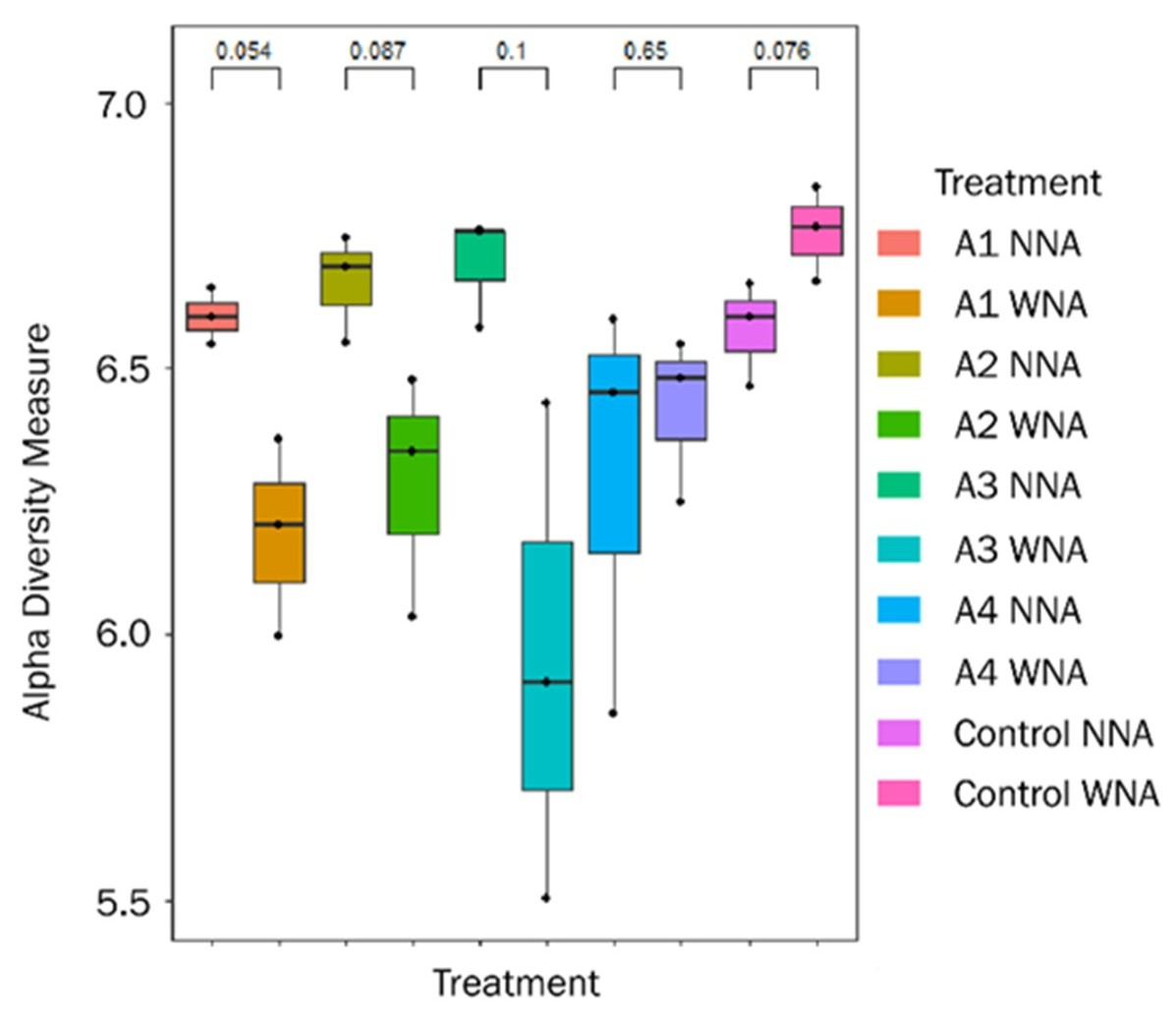

Supplement: S4 Fig — (TIF) [file pone.0259937.s004.tif]

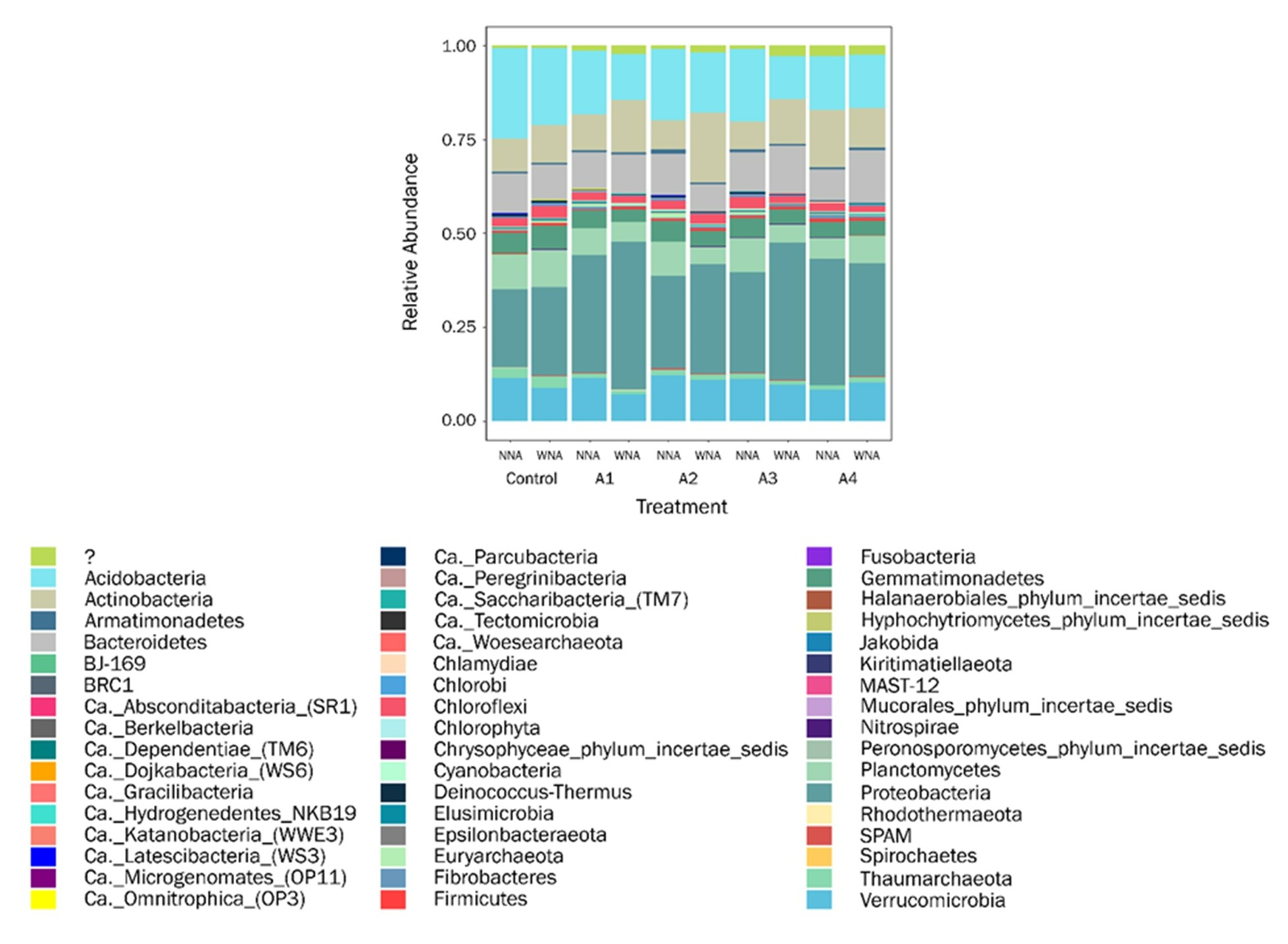

Supplement: S5 Fig — (TIF) [file pone.0259937.s005.tif]

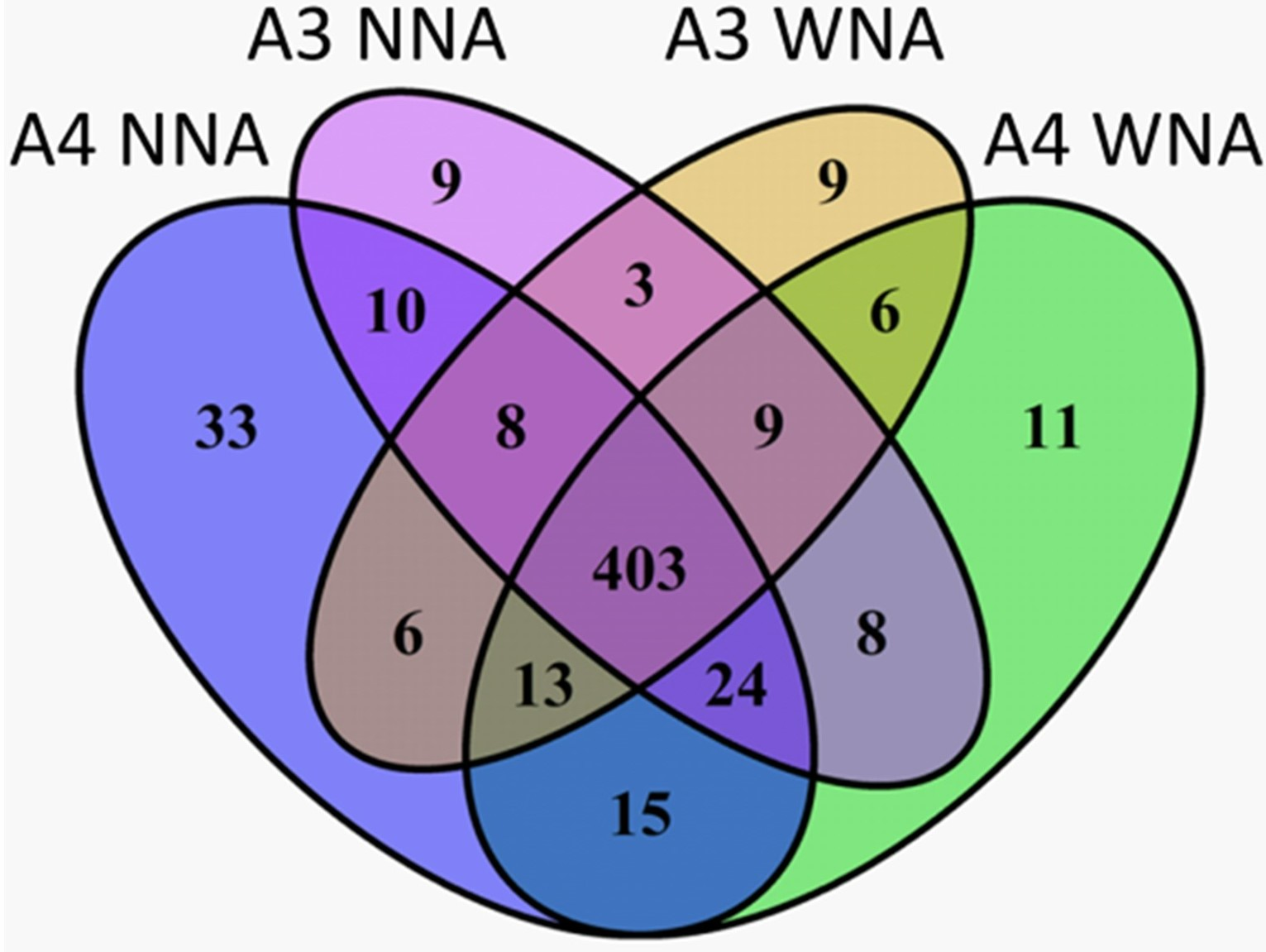

Supplement: S6 Fig — (TIF) [file pone.0259937.s006.tif]

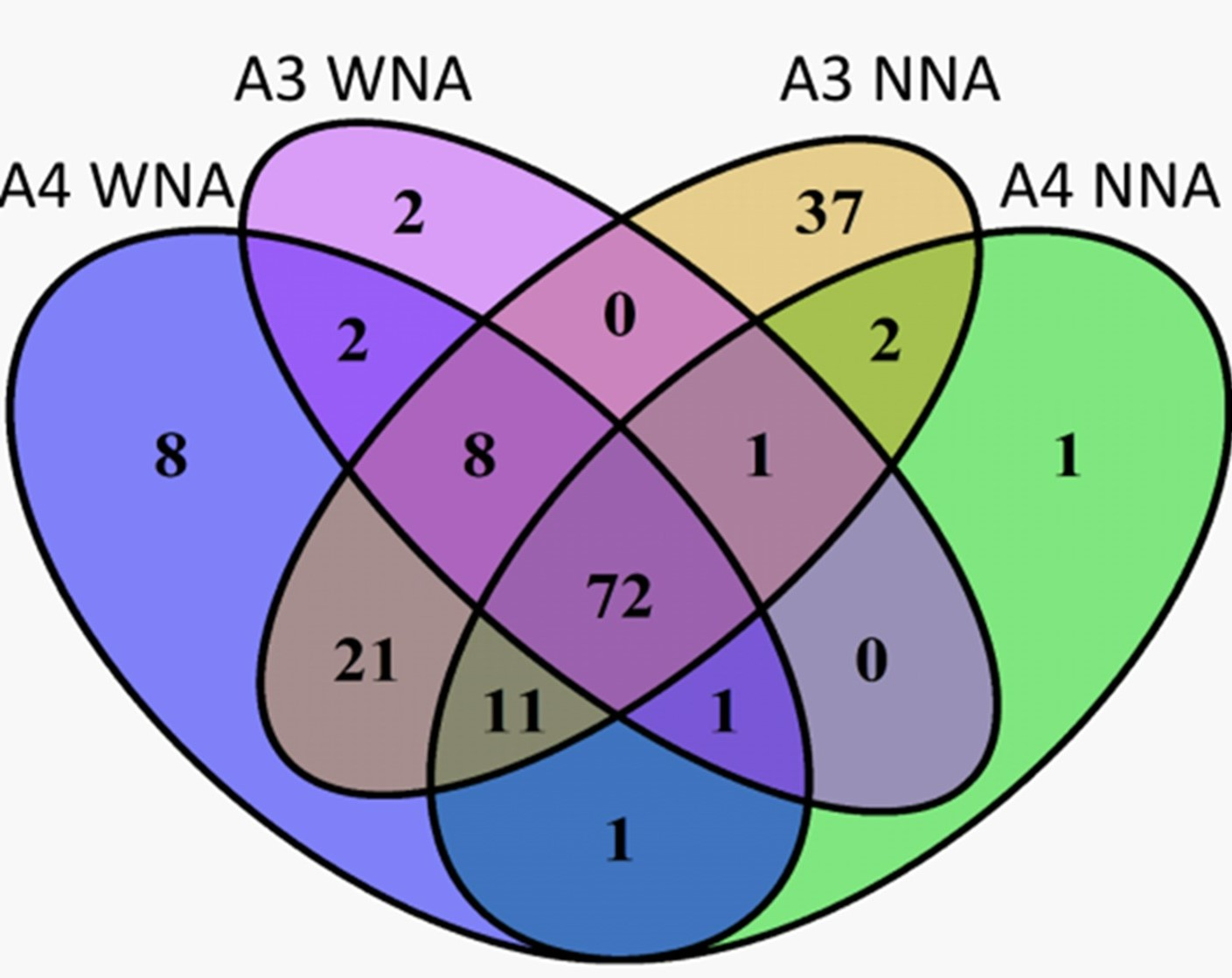

Supplement: S7 Fig — (TIF) [file pone.0259937.s007.tif]

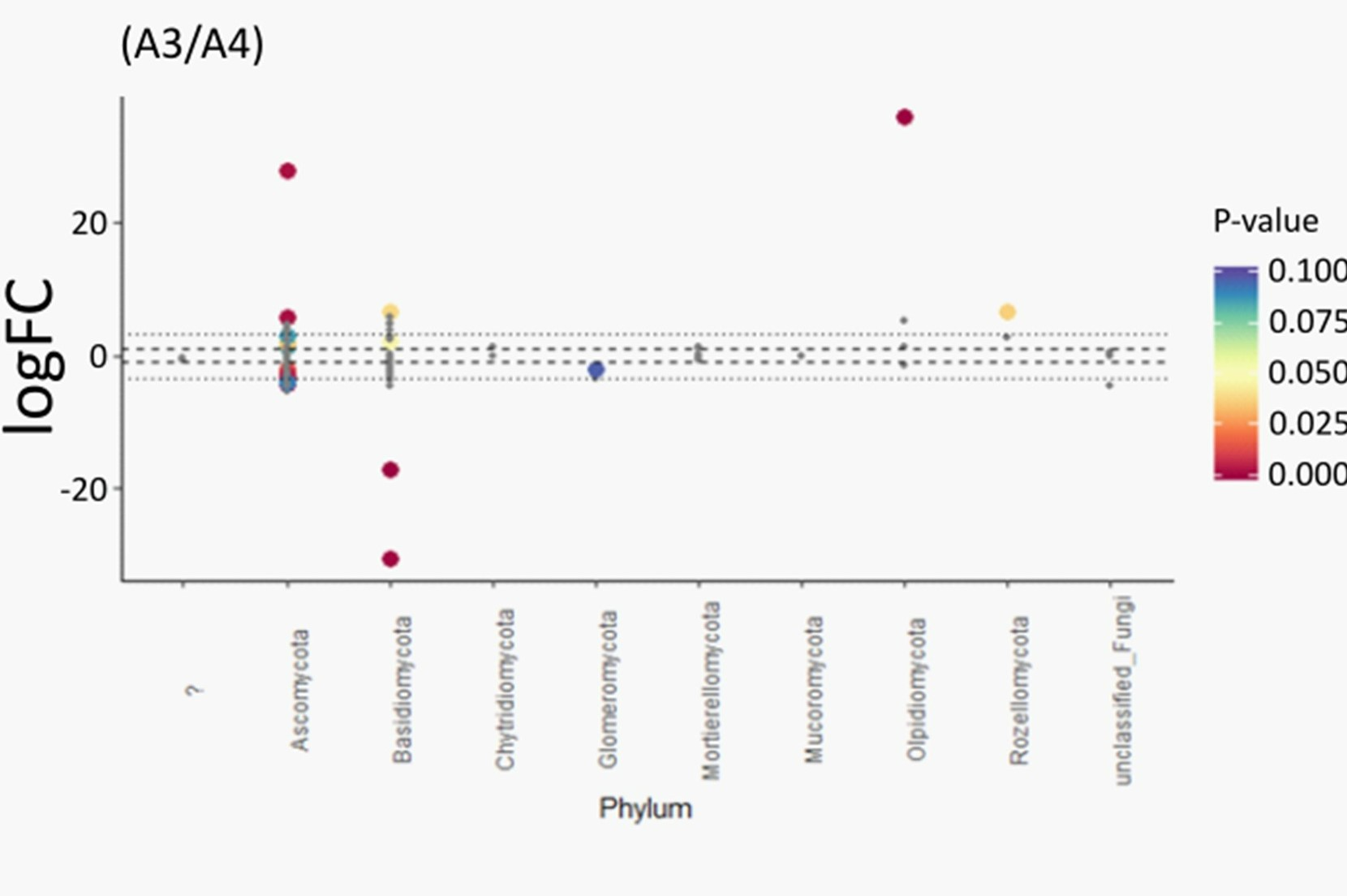

Supplement: S8 Fig — (TIF) [file pone.0259937.s008.tif]

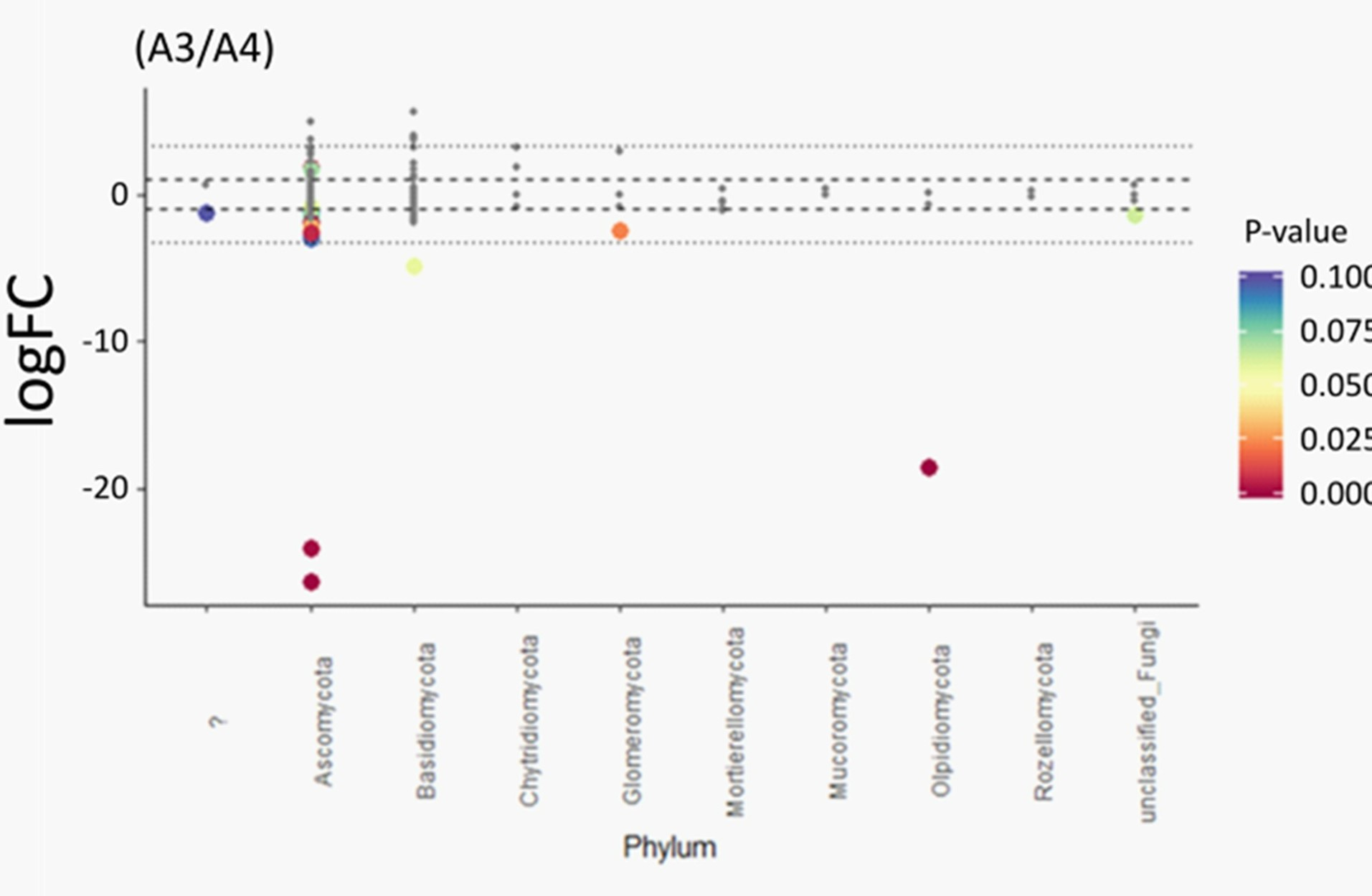

Supplement: S9 Fig — (TIF) [file pone.0259937.s009.tif]

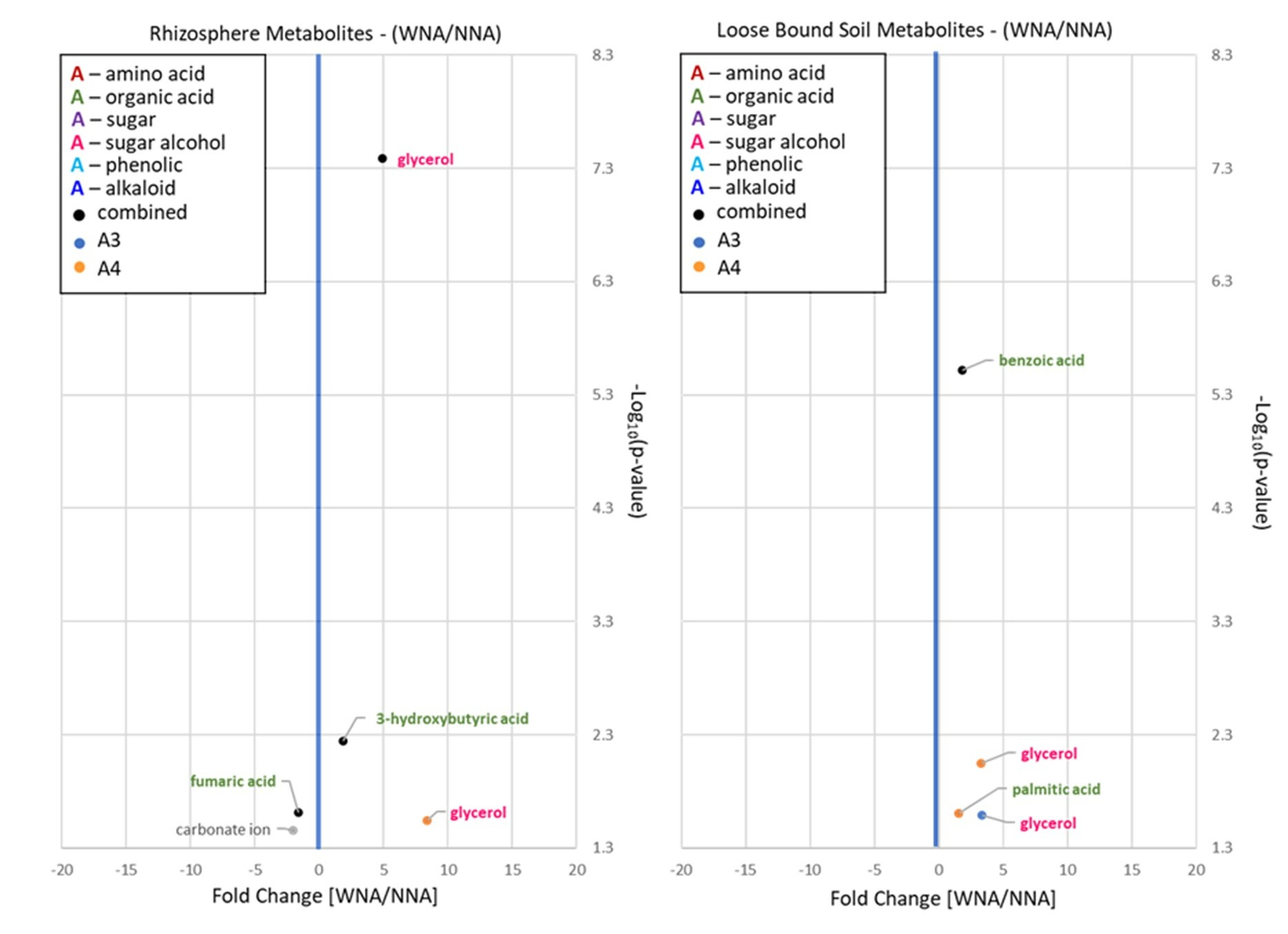

Supplement: S10 Fig — (TIF) [file pone.0259937.s010.tif]

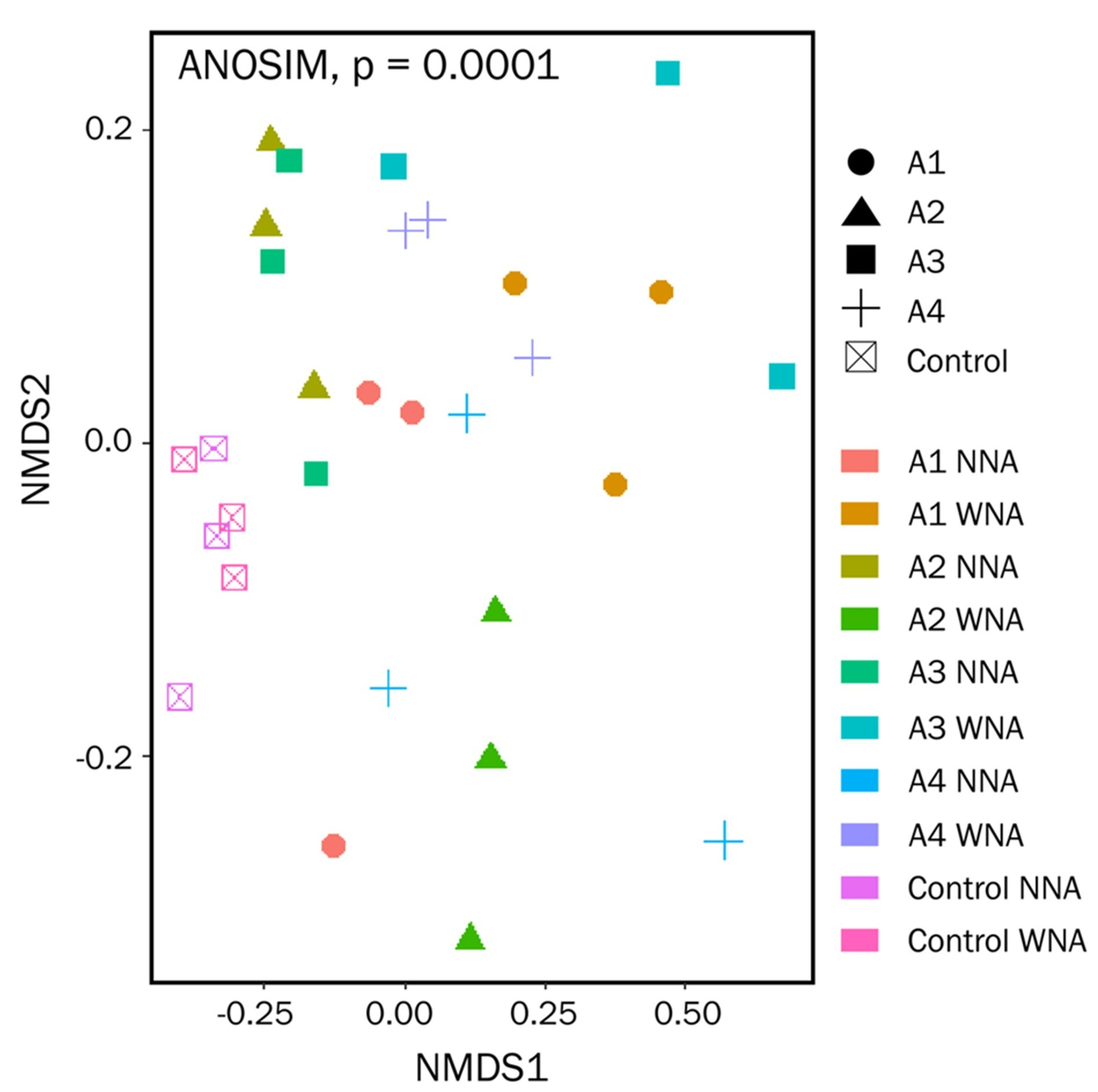

Supplement: S11 Fig — (TIF) [file pone.0259937.s011.tif]

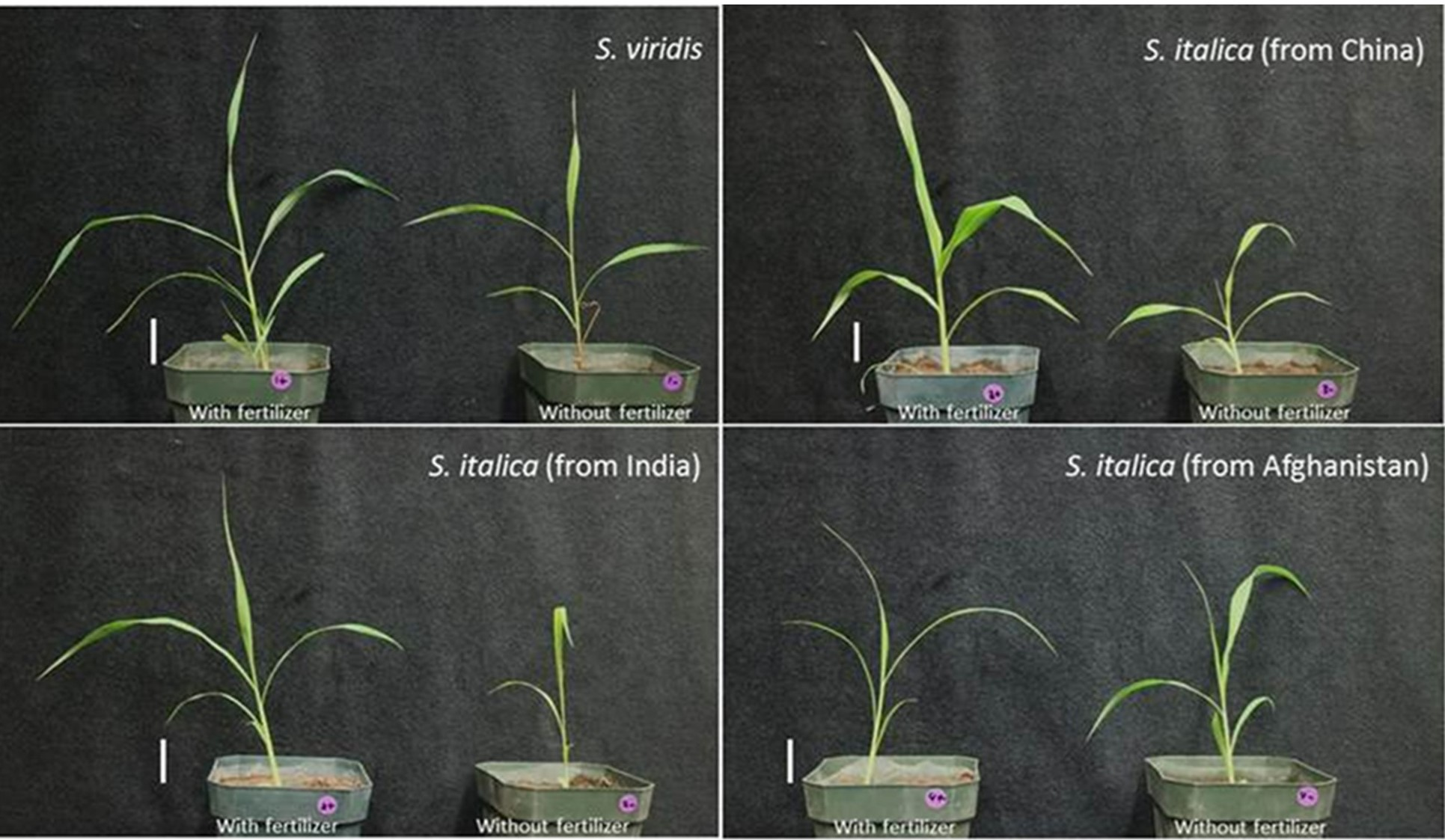

Supplement: S12 Fig — Setaria viridis (A1), and Setaria italica accessions A2–A4 grown with nutrient addition (Left) and no nutrient addition (Right). Vertical scale indicates 1 cm. (TIF) [file pone.0259937.s012.tif]
